# Altered Neural Activity in the Mesoaccumbens Pathway Underlies Impaired Social Reward Processing in *Shank3*‐Deficient Rats

**DOI:** 10.1002/advs.202414813

**Published:** 2025-03-14

**Authors:** Marie Barbier, Keerthi Thirtamara Rajamani, Shai Netser, Shlomo Wagner, Hala Harony‐Nicolas

**Affiliations:** ^1^ Department of Psychiatry New York NY USA; ^2^ Seaver Autism Center for Research and Treatment New York NY 10029 USA; ^3^ Department of Neuroscience New York NY 10029 USA; ^4^ Friedman Brain Institute New York NY 10029 USA; ^5^ Sagol Department of Neurobiology Faculty of Natural Sciences University of Haifa Haifa 31905 Israel; ^6^ Mindich Child Health and Development Institute at the Icahn School of Medicine at Mount Sinai New York NY 10029 USA; ^7^ Present address: Appel Alzheimer's Disease Research Institute, Feil Family Brain and Mind Research Institute Weill Cornell Medicine New York NY 10021 USA

**Keywords:** autism spectrum disorder, dopamine, Phelan‐McDermid syndrome, reward, *Shank3*, social interaction, ventral tegmental area

## Abstract

Social behaviors are crucial for human connection and belonging, often impacted by conditions like Autism Spectrum Disorder (ASD). The mesoaccumbens pathway (ventral tegmental area (VTA) to the nucleus accumbense (NAc)) plays a pivotal role in social behavior and is implicated in ASD. However, the impact of ASD‐related mutations on social reward processing remains insufficiently explored. This study focuses on the *Shank3* mutation, associated with a rare genetic condition and linked to ASD, examining its influence on the mesoaccumbens pathway during behavior, using the *Shank3*‐deficient rat model. Our findings indicate that *Shank3*‐deficient rats exhibit atypical social interactions, associated with altered neuronal activity of VTA dopaminergic and GABAergic neurons and reduced dopamine release in the NAc. Moreover, they demonstrate that manipulating VTA neuronal activity can normalize this behavior, providing insights into the effects of *Shank3* mutations on social reward processing  and identifying a potential neural pathway for intervention.

## Graphical abstract

This study examines the effects of *Shank3* mutation on the mesoaccumbens pathway using a *Shank3*‐deficient rat model. The findings reveal altered social reward processing in these rats, accompanied by changes in dopaminergic and GABAergic neural activity within the ventral tegmental area (VTA)  and dopamine release in the nucleus accumbense (NAc). Modulating VTA activity restores typical social behavior, pionting to a potential neural pathway for intervention.

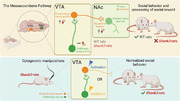

## 1 Introduction

Social behaviors play a pivotal role in shaping our lives and fostering a sense of belonging in society. Deficits in these behaviors are a hallmark feature of several psychiatric and neurodevelopmental disorders, including autism spectrum disorder (ASD).^[^ ^1^ ^]^ The mesoaccumbens reward pathway is involved in regulating certain aspects of social behavior and a growing body of clinical imaging evidence has pointed toward impaired function of this pathway in ASD.^[^ ^2^, ^3^, ^4^, ^5^, ^6^, ^7^, ^8^, ^9^ ^]^ Nevertheless, it remains uncertain whether and in what manner ASD genetic risk factors directly impact the assessment of reward value associated with social cues, as only a limited number of studies have delved into this. Given the lack of effective pharmacological interventions for addressing social behavior deficits, there is a pressing need to elucidate the underlying pathophysiological mechanisms.

The mesoaccumbens pathway connects the ventral tegmental area (VTA) and the nucleus accumbens (NAc), two key brain regions involved in reward processing. The VTA is comprised of diverse cell populations that play distinct roles in motivated behaviors and reward processing, with the majority of these cells being dopaminergic (VTA‐DA) (≈60% of VTA cells) and GABAergic (VTA‐GABA) (35% of VTA cells) neurons.^[^ ^10^, ^11^, ^12^, ^13^, ^14^, ^15^, ^16^, ^17^, ^18^, ^19^ ^]^ VTA‐DA neurons project to several limbic structures including the prefrontal cortex (PFC) and the NAc.^[^ ^13^, ^20^, ^21^, ^22^, ^23^ ^]^ An early neuroimaging study in individuals with ASD showed reduced release of dopamine in the PFC^[^ ^24^ ^]^ and significant increase in DA transporter binding throughout the brain.^[^ ^25^ ^]^ Furthermore, imaging studies involving task performance showed that individuals with ASD display a reduction in phasic striatal DA events evoked by social stimuli.^[^ ^8^, ^26^ ^]^ Together, these findings suggest that functional changes in the dopaminergic system may be implicated in the pathophysiology of ASD.

One notable genetic condition associated with ASD is Phelan‐McDermid syndrome (PMS).^[^ ^27^, ^28^ ^]^ This rare disorder is caused by mutations or deletions in the *SHANK3* gene, which encodes for a critical scaffolding protein in the core of the postsynaptic density.^[^ ^29^, ^30^, ^31^, ^32^, ^33^, ^34^, ^35^ ^]^ Individuals with PMS present with a wide range of developmental, cognitive, and medical abnormalities.^[^ ^36^, ^37^ ^]^ Those include generalized developmental delay with intellectual disability of variable severity, absent or delayed speech, hypotonia, motor skill deficits, seizures, gastrointestinal problems, renal malformations, and non‐specific dysmorphic features.^[^ ^37^, ^38^, ^39^ ^]^ Psychiatric symptoms are prominent in a portion of individuals with PMS, including atypical bipolar disorder, catatonia, attention‐deficit hyperactivity disorder,^[^ ^40^, ^41^, ^42^, ^43^ ^]^ and ASD.^[^ ^36^, ^37^ ^]^ Approximately 63% of individuals with PMS meet the Diagnostic and Statistical Manual of Mental Disorder‐5 criteria for ASD^[^ ^37^ ^]^ and up to 2% of individuals with ASD have *SHANK3* haploinsufficiency.^[^ ^44^, ^45^ ^]^

To date, there have been no clinical studies examining the involvement of the mesoaccumbens pathway in the PMS phenotype or investigating how social and non‐social reward processing may be affected. Despite the lack of clinical studies, the creation and characterization of rodent models with a *Shank3* gene mutation, have consistently demonstrated that *Shank3* deficiency has a detrimental effects on synaptic transmission and plasticity within several brain regions including the striatum and hippocampus; two brain regions that receive DA projections from the VTA.^[^ ^28^, ^34^, ^46^, ^47^, ^48^, ^49^, ^50^, ^51^, ^52^ ^]^ Deficits in synaptic transmission were also observed in the VTA of mice following early postnatal down expression of the Shank3 protein within the VTA. Specifically, Bariselli and colleagues, demonstrated that down expression of Shank3 in the VTA of mice impairs the maturation of excitatory synapses onto VTA‐DA and VTA‐GABA neurons, leading to a decrease in VTA‐DA and an increase in VTA‐GABA neural activity.^[^ ^47^ ^]^

Expanding on these results, in our current study we asked what are the consequences of *Shank3* deficiency on real‐time activity of VTA neurons, as well as the release of DA in the NAc during social and non‐social behaviors. To address our questions, we opted for a rat model for *Shank3* deficiency, given the unique advantages rats offer compared to mice, such as a more complex social behavior, higher level of social motivation, larger brain size, closer physiological similarity to humans, and their suitability for pharmacological testing, making them an invaluable tool for studying autism‐related genes and translating findings to clinical applications. Specifically, we used a previously validated *Shank3*‐deficient rat model,^[^ ^49^ ^]^ which carries a mutation in the *Shank3* gene that is similar to a human *SHANK3* mutation.^[^ ^53^ ^]^ This model allows us to investigate the consequences of *Shank3* deficiency in the entire organism during early developmental stages, a condition that closely resembles the patient's condition. We utilized fiber photometry tools coupled with calcium or DA sensors to accurately track the activity of DAand GABAneurons in the VTA, as well as the release of DA in the NAc, in rats during their participation in behavioral tasks. Ultimately, we employed optogenetic techniques to investigate whether altering the activity of VTA neurons could bring about changes in the behavioral phenotype. This allowed us to draw causality between activity of the mesoaccumbens pathway and the observed behavioral phenotype.

## 2 Results

### 2.1 *Shank3*‐Deficient Rats Exhibit an Atypical Social Interaction that is Associated with Impaired VTA‐DA Neural Activity

**Figure 1:**
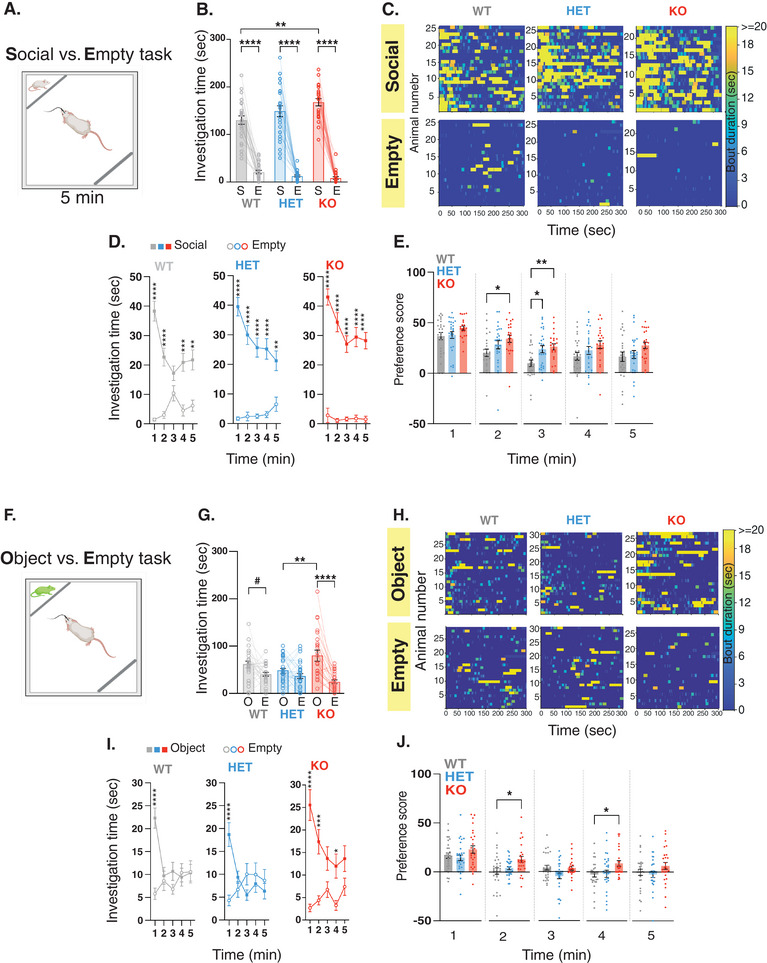
*Shank3*‐deficient rats show an atypical behavior when presented with a social or object stimuli. A) Behavioral outline for the Social versus Empty task at Satiety. Rats encounter either a confined novel same‐sex juvenile or an empty compartment. B) Mean total investigation time of the social stimulus or the empty compartment. C) Heat maps depicting the investigation time of individual rats during the 5‐minute test period. Each row represents one rat. D) Mean total investigation time for the social stimulus and the empty compartment averaged in 1‐minute intervals over the 5‐minute test period. E) Differences in mean investigation time between the social stimulus and the empty compartment calculated for each minute. B‐E: WT, n = 25; HET, n = 26; KO, n = 23. F) Behavioral outline for the Object versus Empty task. Rats encounter either a confined, moving toy‐rat or an empty compartment. G‐J) Similar to B‐E, but show behavior during the Object versus Empty task at Satiety. G‐J: WT, n = 29; HET, n = 30; KO, n = 27. ****p <.0001, ***p <.001, **p <.01, *p <.05, post hoc tests following the main effect. All error bars represent SEM. Detailed statistical data are provided as a Data file in Table S1 (Supporting Information).

To examine the effects of *Shank3* mutation on social behavior and the associated neural activity within the VTA, we first conducted a comprehensive assessment of the *Shank3*‐deficient rats’ behavior using the Social versus Empty task. In this task, subject rats were presented with two compartments, one that contained a social stimulus (juvenile rat) and the other that was kept empty (**Figure** **1**A). We found that wildetype (WT) rats and their *Shank3*‐Heterozygous (Het) and *Shank3*‐Homozygous/Kockout (KO) littermates all spent more time investigating the compartment containing a same‐sex juvenile rat stimulus, compared to the empty compartment (Figure 1B; Figure S4A, Supporting Information). At first glance, this outcome may be misconstrued to suggest that *Shank3*‐deficient rats exhibit social behavior that is similar to their WT littermates. However, *Shank3*‐KO rats exhibited distinctly elevated time interacting with the social stimuli, when compared to their WT littermates (Figure 1B). They also exhibited significantly higher engagement in long‐bouts of social investigation (>19 s), previously shown to be more associated with social interaction^[^ ^54^ ^]^ (Figure S4B, Supporting Information, upper right panel), and significantly lower engagement with the empty compartment, regardless of the bout length (Figure S4B, Supporting Information, lower panels), with no change in the total number of bouts (Figure S4C, Supporting Information). A detailed analysis of the overall interaction characteristics during the 5‐minute testing period further re‐affirmed that *Shank3*‐deficient rats exhibit an atypical social behavior. WT rats showed an increase in transitioning between the stimuli (Figure S4D, Supporting Information, upper panels), while *Shank3*‐HET and KO rats showed no change in the transition rate over the 5‐minutes period of testing (Figure S4D, Supporting Information, middle and lower panels, respectively). This atypical behavior was accompanied with a distinct pattern of investigation across the 5 min of testing. Specifically, WT rats exhibited a substantial decline in their engagement with the social stimuli at the second minute of testing, to the extent that they displayed no preference between the social and empty compartments at the third minute (Figure 1C,D, left panels). *Shank3*‐Het and *Shank3*‐KO rats, however, continued to interact with the social stimulus and exhibited almost no interest in the empty compartment throughout the entire 5 min of testing (Figure 1C,D, middle and right, respectively). These differences in stimuli preference between genotypes were evident through the statistically significant variations in the preference score during the second and third minutes of testing (Figure 1E). Critically, in a comparable paradigm, where we introduced an object (a moving rat toy) instead of a social stimulus (Figure 1F, Object versus Empty), both WT and *Shank3*‐KO rats exhibited a preference for the novel object (Figure 1G; Figure S4E, Supporting Information), albeit to a lesser degree compared to their preference to the social stimulus in the Social versus Empty task. However, when analyzing the investigation time across the 5‐minutes testing period, we found that although rats from all three genotypes showed a strong preference to the object stimulus during the 1st minute, WT and *Shank3*‐Het rats quickly lost interest in investigating the object following the first minute (Figure 1H,I, left and middle panels, respectively), while *Shank3*‐KO rats continued to spend more time at the object compartment (Figure 1H,I, right panels and Figure 1J). We observed no significant differences in the cumulative bout length (Figure S4F, Supporting Information), bout number (Figure S4G, Supporting Information), or transition pattern (Figure S4H, Supporting Information) on the Object versus Empty task. Together, these findings suggest that *Shank3*‐KO rats demonstrate an atypical increase in interaction when faced with a single stimulus choice, regardless of whether it involved social or non‐social stimuli.

**Figure 2:**
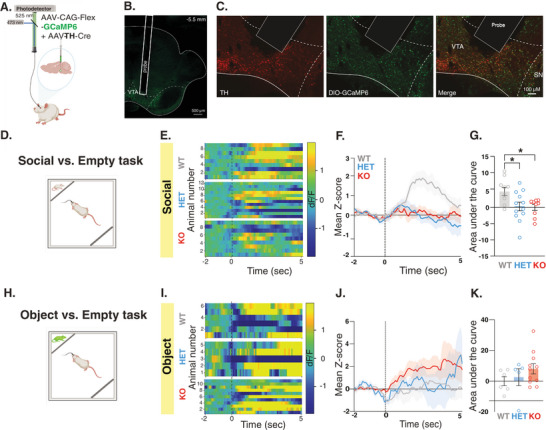
*Shank3*‐deficient rats show impaired VTA‐DA neural activity during interaction with a social but not an object stimulus. A) A schematic of viral injection and fiber photometry setup in the VTA. B) A coronal section of the VTA showing the viral injection and the optical fiber placement (10X magnification; scale bar  = 500 µm). C) Immunohistochemistry staining of a sample VTA section showing TH expression (left panel, red), GCaMP6 (viral) expression (middle panel, green), and overlap (right panel), along with fiber placement (10X magnification; scale bar  = 100 µm). D) Behavioral outline for the Social versus Empty task at Satiety. Rats encounter either a confined novel same‐sex juvenile or an empty compartment. E‐G) GCaMP6 Fiber photometry recordings of VTA‐DA neurons during the Social versus Empty task. WT, n = 10; HET, n = 12; KO, n = 9. E) Heat maps illustrate the change in fluorescence signals (dF/F) from 2 s before to 5 seconds after each social interaction bout. Each row corresponds to one animal and comprises an average of signals from all interaction bouts during the 5‐minute testing period. F) Average standardized VTA‐DA photometry responses aligned to investigation onset of the social stimulus G) The area under the curve, calculated from average standardized traces in (F). H‐K) Similar to D‐G, but shows behavioral data and fiber photometry recording during the Object versus Empty task at Satiety. WT, n =11 HET, n = 13; KO, n = 11. All error bars represent SEM. Detailed statistical analysis is provided as a Data file in Table S1 (Supporting Information).

To record neural activity of VTA neurons during behavior, we employed an in vivo fiber photometry calcium imaging approach in conjunction with a genetically encoded calcium indicator (GCaMP). This technique provides a highly sensitive tool to record cell‐type specific neuronal activity in behaving animals within deep brain structures,^[^ ^55^, ^56^ ^]^ such as the VTA.^[^ ^57^ ^]^ To specifically target VTA‐DA neurons, we used a dual viral approach where one virus expressed a Cre‐dependent GCaMP6 (AAV9‐CAG‐FLEX‐GCaMP6m) and a second virus expressed Cre recombinase under the control of the tyrosine hydroxylase (TH) promoter, a common promoter used to target DA neurons^[^ ^58^ ^]^ (AAV9.rTH.PI.Cre.SV40) (**Figure** **2**A,B). Three weeks following viral expression, we recorded GCaMP6 fluorescent signals during the Social versus Empty task (Figure 2C). We found a significant increase of calcium signals in WT rats during social interaction (Figure 2D‐G), reflecting an increase in VTA‐DA neural activity that is consistent with previous reports in mice.^[^ ^46^, ^47^, ^57^, ^59^ ^]^ In contrast, despite displaying seemingly active and even elevated interaction with the social stimuli (Figure S5A‐B, Supporting Information), *Shank3*‐Het and KO rats did not exhibit an increase in VTA‐DA neural activity during social interaction (Figure 2D‐G), and showed a comparable pattern of activity when approaching the object on the Object versus Empty task (Figure 2H‐K; Figure S5C‐D, Supporting Information). These results raise the possibility that *Shank3*‐deficient rats do not appropriately process the rewarding value of social interactions, due to the lack of VTA‐DA neuronal activity, hence are not getting satisfaction out of it. Furthermore, they propose that the increased interaction with the object observed in *Shank3*‐KO rats during the object versus empty task is not due to alterations in VTA‐DA neuron activity, but rather to disruptions in other neural activities.

### 2.2 *Shank3*‐Deficient Rats Show Deficits in Shifting their Behavior Based on the Value of the Social and Non‐Social Rewards

**Figure 3:**
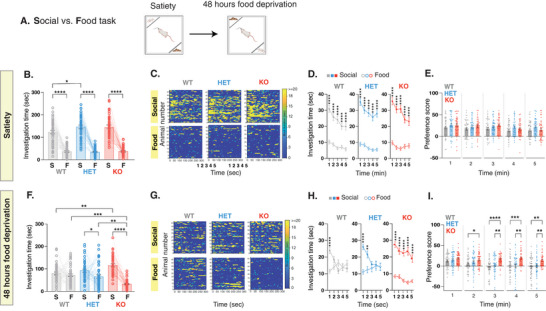
*Shank3*‐deficient rats show deficits in shifting their behavior based on the value of the social and non‐social rewards. A) Behavioral outline for the Social versus Food task at satiety and after 48 hours of food deprivation. Rats encounter either a confined novel same‐sex juvenile or a food‐containing compartment. B) Mean total investigation time of the social stimulus or the food‐containing compartment at satiety. C) Heat maps depicting the investigation time of individual rats during the 5‐minute test period. Each row represents one rat. D) Mean total investigation time for the social stimulus and the food‐containing compartment averaged in 1‐minute intervals over the 5‐minute test period. E) Differences in mean investigation time between the social stimulus and the food‐containing compartment for each minute. F‐I) Similar to B‐E, but show behavior after 48 h of food deprivation. WT, n = 47; HET, n = 47; KO, n = 41. ****p <.0001, ***p <.001, **p <.01, *p <.05, post hoc tests following the main effect. All error bars represent SEM. Detailed statistical data are provided as a Data file in Table S1 (Supporting Information).

To investigate if *Shank3*‐deficient rats can process the value of a rewarding stimuli and alter their behavior based on the change in reward value of the presented stimuli, we used the Social versus Food task (**Figure** **3**A). This task models a more naturalistic settings where animals are faced with more than one rewarding choice.^[^ ^54^, ^60^ ^]^ Specifically, rats are presented concurrently with a social (same‐sex juvenile rat) and non‐social rewarding stimuli (food), each at opposing compartment while at satiety and then tested again on the same paradigm following food deprivation. As social interaction is typically more rewarding than food in caloric satiation state, rats are expected to spend more time investigating the social stimulus than food. However, when food‐deprived, the value of food increases, as a result rats are expected to shift their behavior accordingly.^[^ ^54^, ^60^ ^]^ At satiety, both *Shank3*‐deficient rats and their WT littermates exhibited a greater inclination towards engaging with the social stimulus rather than food (Figure 3B‐E; Figure S6A, Supporting Information left panel) with *Shank*3‐HET rats showing a slightly increased interest in the social stimulus (Figure 3B). Also, number of bouts (Figure S6B, Supporting Information) and total investigation time across bouts (Figure S6C, Supporting Information) were comparable between genotypes. While *Shank3*‐deficient rats displayed no noticeable change in transitions, compared to WT littermates that demonstrated fewer transitions over time (Figure S6D, Supporting Information), overall we observed no major differences in behavior between genotypes on this task. However, when tested again on the same Social versus Food task after 48 h of food deprivation, WT rats shifted their behavior to spend an equal time investigating both stimuli, while *Shank3*‐KO rats continued to spend significantly more time investigating the social stimulus (Figure 3F; Figure S6A, Supporting Information, right panel). Compared to their WT littermates, *Shank3*‐KO rats also displayed a significantly higher number and cumulative bout length toward the social stimulus, and a lower number of bouts and cumulative bout length towards the food (Figure S6E‐F, Supporting Information) with no substantial difference in the transition pattern between the two stimuli (Figure S6G, Supporting Information). The pattern of increased investigation exhibited by the *Shank3*‐deficient rats was clearly sustained across the 5 min of testing (Figure 3G‐I). To rule out the possibility that the diminished interest of *Shank3*‐deficient rats in interacting with food is due to reduced appetite, we subjected the rats to a 48‐hour food deprivation period. Afterward, we gave them unrestricted access to food without any additional stimuli in the arena or while presenting them with either a novel juvenile or a moving toy object. *Shank3*‐deficient rats consumed food at normal levels when no stimuli were introduced into the testing arena and when a moving object was present (Figure S7A‐B, Supporting Information, respectively). However, when presented with a social stimulus, *Shank3*‐deficient rats displayed a significant decrease in food consumption (Figure S7C, Supporting Information). Together, these findings suggest that only when presented with a social stimulus, *Shank3*‐deficient rats show impairment in adjusting their behavior based on the value of the presented stimuli.

**Figure 4:**
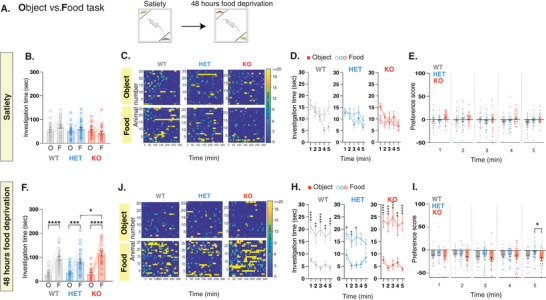
*Shank3‐*deficient rats exhibit a typical behavior when presented with an object stimulus. A) Behavioral outline for the Object versus Food task at satiety and after 48 h of food deprivation. Rats encounter either a confined moving rat toy or a food‐containing compartment. B) Mean total investigation time of the object stimulus or the food‐containing compartment at satiety. C) Heat maps depicting the investigation time of individual rats during the 5‐minute test period. Each row represents one rat. D) Mean total investigation time for the object stimulus and the food‐containing compartment averaged in 1‐minute intervals over the 5‐minute test period. E) Differences in mean investigation time between the object stimulus and the food‐containing compartment for each minute. F‐I) Similar to B‐E, but show behavior after 48 hours of food deprivation. WT, n = 25; HET, n = 26; KO, n = 23. ****p <.0001, ***p <.001, **p <.01, *p <.05, post hoc tests following the main effect. All error bars represent SEM. Detailed statistical data are provided as a Data file in Table S1 (Supporting Information).

To further substantiate our findings, we investigated the behavior of the rats using the Object versus Food task. In this task, rats were simultaneously exposed to both an object and food when they were satiated (**Figure** **4**A). This setup ensured that the object and food were of equal or no reward value. *Shank3*‐deficient rats and their WT littermates showed no preference to either the object or food compartments at satiety (Figure 4B‐E; Figure S8A, Supporting Information, left panel). Furthermore, there were no substantial difference across genotypes in the number of bouts (Figure S8B, Supporting Information), in the cumulative bout investigation time (Figure S8C, Supporting Information), or in the transition pattern (Figure S8D, Supporting Information). Importantly, similar to their WT littermates, *Shank3*‐*deficient* rats continued to exhibit a typical behavior also following food deprivation, showing preference to food over object (Figure 4F‐I), as well as similar number of bouts and cumulative bout investigation time (Figure S8E,F, Supporting Information, respectively), and transition patterns (Figure S8G, Supporting Information). Taken together, our findings suggest that the atypical behavior observed in the *Shank3*‐deficient rats pertain specifically to their ability to adjust their behavior based on the rewarding values of two competing stimuli. These impairments are context‐specific, becoming apparent when confronted with social stimulus alongside another stimulus of competing value.

### 2.3 *Shank3*‐Deficient Rats Show Impairment in VTA‐DA Neural Activity During Social but not Food or Object Interaction

**Figure 5:**
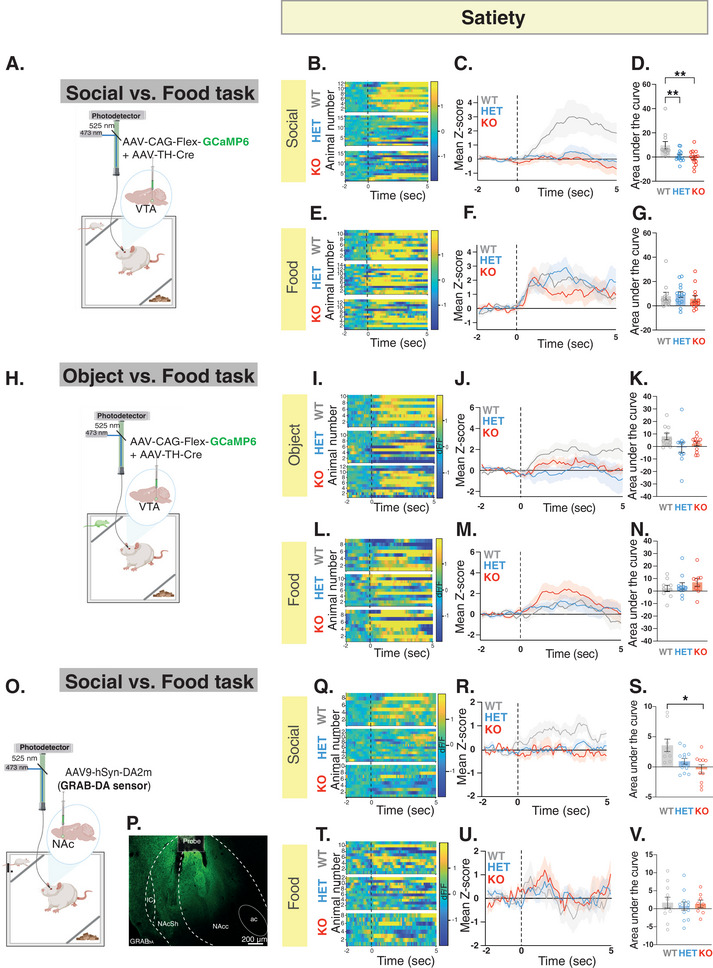
*Shank3*‐deficient rats show impaired VTA‐DA neural activity during interaction with a social but not an object or food stimulus. A) A schematic of viral injection and fiber photometry setup in the VTA. B‐G) GCaMP6 fiber photometry recordings of VTA‐DA neurons during the Social versus Food task at Satiety. B. Heat maps illustrate the change in fluorescence signals (dF/F) from 2 seconds before to 5 seconds after each interaction bout for the social stimulus. Each row corresponds to one animal and comprises an average of signals from all interaction bouts during the 5‐minute testing period. C. Average standardized VTA‐DA photometry responses aligned to investigation onset of the social stimulus. D. The area under the curve, calculated from average standardized traces in (C). E‐G. Similar to B‐D, but shows fiber photometry recording when the rats explore the food. WT, n = 14; HET, n = 14; KO, n = 16. H‐N) Similar to A‐G, but shows behavioral data and fiber photometry recording during the Object versus Food task at Satiety. WT, n = 10; HET, n = 12; KO, n = 13. O) A schematic of viral injection and fiber photometry setup in the NAc. P) Immunohistochemistry staining of a sample NAc section showing GRAB_DA_ sensor expression (in green, 10X magnification; scale bar  = 200 µm). Q‐V) Similar to B‐G, but show fiber photometry recording of GRAB_DA_ sensor in the NAc during the Social versus Food task at Satiety. WT, n = 10; HET, n = 14; KO, n = 10. **p <.01, *p <.05, post hoc tests following the main effect. All error bars represent SEM. Detailed statistical data are provided as a Data file in Table S1 (Supporting Information).

To examine if the observed atypical behavior is correlated with changes in VTA‐DA neural activity, we recorded neural activity during both the Social versus Food (**Figure** **5**A; Figure S9, Supporting Information) and Object versus Food tasks (Figure 5H) during the satiety state, using GCaMP6 signals for recording, while simultaneously confirming that the previously observed behavioral phenotype is replicated in this cohort of rats (Figure S10A‐H, Supporting Information). Our analysis revealed that VTA‐DA neural activity increased during social interaction in WT rats, whereas no such increase was observed in *Shank3*‐Het or *Shank3*‐KO rats (Figure 5B‐D). Notably, this lack of response was observed only during interaction with a social stimulus, as VTA‐DA neural activity was elevated in all three genotypes when rats explored the food compartment (Figure 5E‐G). Recording VTA‐DA activity during the Social versus Food Task after food deprivation showed no significant differences between genotypes during either social or food interactions (Figure S11, Supporting Information). Importantly, the absence of differences does not contradict our findings of altered neural activity in *Shank3*‐deficient rats during satiety. Instead, it is attributed to the low activity levels observed in WT rats, potentially reflecting a reduced rewarding value under these conditions (Figure S11, Supporting Information). Finally, when recording during the Object versus Food task, we observed no statistically significant differences across genotypes, regardless of the stimulus (Figure 5I‐N). These results reaffirm our earlier findings that, although *Shank3*‐deficient rats spend more time engaged in social interaction, their distinctive social interaction characteristics coupled with the absence of an increase in VTA‐DA neural activity, suggest ineffective processing of the rewarding aspect of social interaction. Furthermore, these findings emphasize that the lack of enhanced VTA‐DA activity in behaving rats is specifically notable during social interaction.

### 2.4 *Shank3*‐Deficient Rats Show Impaired DA Release in the NAc During Social Interaction

To investigate whether the impaired behavior and the VTA‐DA neural activity deficits are also associated with disrupted DA release in the NAc during social interaction, we utilized the G protein‐coupled receptor [GPCR]‐activation‐based‐DA (GRABDA) sensor^[^ ^61^ ^]^ in combination with fiber photometry to examine changes in NAc‐DA levels during social interaction in an independent cohort of rats (Figure S12A‐D, Supporting Information). Specifically, we injected the AAV9‐hSyn‐DA2m (DA4.4) into the NAc and implanted an optic fiber just above the injection site (Figure 5O,P). Three weeks following viral expression, we recorded the fluorescent signals of GRAB_DA_ during the Social versus Food task when animals were satiated. Our findings showed that in WT rats, social interaction was accompanied by elevated DA release in the NAc, whereas this effect was not observed in the *Shank3*‐*deficient* rats (Figure 5Q‐S). Less increase was associated with exploration of the food compartments in all genotypes (Figure 5T‐V). Together, these results suggest that the reduced neural activity in the VTA‐DA neurons of *Shank3*‐deficinet rats aligns with a decrease in DA release in downstream VTA regions, notably the NAc, specifically during social interaction.

### 2.5 *Shank3*‐*Deficient* Rats Show Abnormal Increase in VTA‐GABAergic Neural Activity During Social Interaction

**Figure 6:**
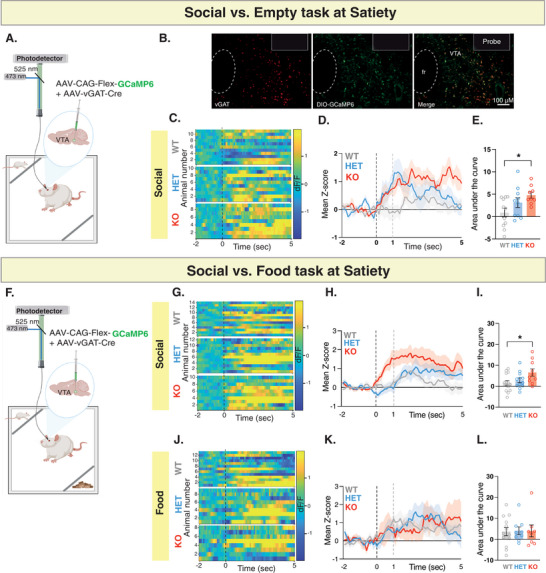
*Shank3*‐deficient rats show abnormal increase in VTA‐GABA neural activity during social interaction. A) A schematic of viral injection and fiber photometry setup in the VTA. B) Immunohistochemistry staining of a sample VTA section showing vGAT expression (left panel, red), GCaMP6 (viral) expression (middle panel, green), and overlap (right panel), along with fiber placement (10X magnification; scale bar  = 100 µm). C) Heat maps illustrate the change in GCaMP6 fluorescence signals (dF/F) from 2 s before to 5 s after each interaction bout for the social stimulus at Satiety. Each row corresponds to one animal and comprises an average of signals from all interaction bouts during the 5‐minute testing period. D) Average standardized VTA‐GABA photometry responses aligned to investigation onset of the social stimulus. E) The area under the curve, calculated from average standardized traces in (D). WT, n = 11; HET, n = 10; KO, n = 9. F‐L) Similar to A‐E, but show fiber photometry recording during the Social versus Food task at Satiety. WT, n = 14; HET, n = 12; KO, n = 10. *p <.05. All error bars represent SEM. Detailed statistical analysis is provided as a Data file in Table S1 (Supporting Information).

To investigate the impact of *Shank3* mutation on neural activity of VTA‐GABA neurons during social interaction, we injected into the VTA the AAV9‐CAG‐FLEX‐GCaMP6m virus, which was responsible for expressing GCaMP6 in a Cre‐dependent manner and the rAAV‐hVGAT1‐Cre‐WPRE‐hGH polyA virus, which expressed the Cre recombinase under the control of the vesicular GABA transporter (vGAT) promoter, commonly utilized to target GABAergic neurons^[^ ^62^ ^]^ (**Figure** **6**A,B; Figure S13, Supporting Information). Our behavioral findings from this independent cohort of rats showed a similar pattern of behavior across the 5‐minute testing period (Figure S14A,B, Supporting Information). Our recording revealed that while WT rats exhibited a slight increase in VTA‐GABA activity when approaching the social stimulus, both *Shank3*‐Het and *Shank3*‐KO rats displayed a significantly higher increase in VTA‐GABA activity (Figure 6C‐E). During the Social versus Food task (Figure 6F; Figure S14C‐F, Supporting Information), we observed a comparable trend of increased VTA‐GABA activity, noticeable only when the rats approached the social, but not the food stimulus (Figure 6 G‐I and Figure 6J‐L, respectively). Similar to the absence of genotype differences in VTA‐DA activity after food deprivation, VTA‐GABA neural activity also showed no differences across genotypes under the same conditions (Figure S15, Supporting Information). Finally, we found no differences in VTA‐GABA activity patterns between genotypes in either the Object versus Empty task (Figure S16A‐F, Supporting Information) or Object versus Food task (Figure S17A‐K, Supporting Information), ruling out the possibility that changes in VTA‐GABA neuron activity contribute to the increased interaction with the object seen in the Object versus Empty task. Taken together, these findings demonstrate that *Shank3*‐deficient rats exhibit an atypical increase in VTA‐GABA neural activity that is specific to social interaction and not generalized across all stimuli.

### 2.6 Optogenetic Activation of VTA‐DA Neurons During Social Interaction Normalizes the Atypical Behavior in *Shank3*‐KO Rats

**Figure 7:**
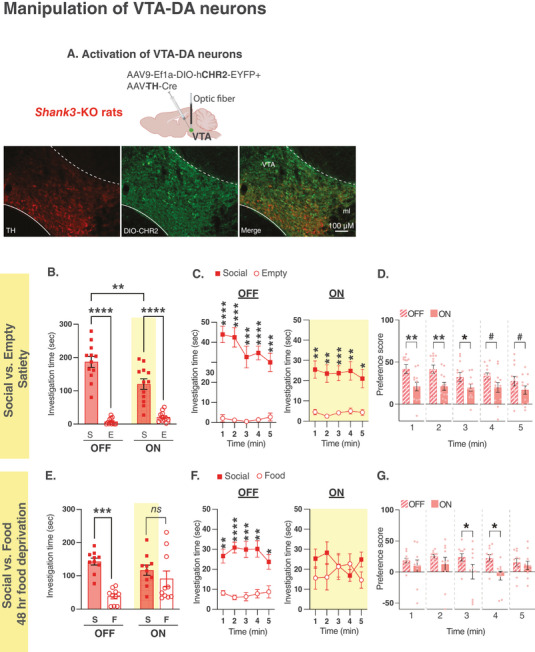
Optogenetic activation of VTA‐DA neurons improves the atypical behavior observed in *Shank3*‐deficient rats. A) A schematic of viral injection and optical fibers placement in the VTA of *Shank3*‐KO rats (Top) and immunohistochemistry staining (bottom) of a sample VTA section showing TH expression (left panel, red), CHR2 (viral) expression (middle panel, green) and overlap (right panel). 10X magnification; scale bar  = 100 µm. B) Mean total investigation time of the social stimulus or the empty compartment during the non‐activation (OFF) or activation (ON) of the VTA‐DA neurons in *Shank3*‐KO rats (n = 12) when the rats approach the social stimulus at Satiety. C) Mean total investigation time for the social stimulus and the empty compartment averaged in 1‐minute intervals over the 5‐minute test period in *Shank3*‐KO rats. D) Differences in mean investigation time between the social stimulus and the empty compartment for each minute in *Shank3*‐KO rats. E‐G) Similar to B‐D, but show behavioral data during the Social versus Food test at 48 hours of food deprivation in *Shank3*‐KO rats (KO, n = 10). ****p <.0001, ***p <.001, **p <.01, *p <.05, post hoc tests following the main effect. All error bars represent SEM. Detailed statistical data are provided as a Data file in Table S1 (Supporting Information).

In light of our findings, we next tested whether optogenetic activation of VTA‐DA neurons could normalize the atypical behavioral phenotype in *Shank3*‐KO rats to a level comparable to that of their WT littermates. To selectively activate VTA‐DA neurons, we injected the AAV‐TH‐Cre virus along with the AAV9‐Ef1a‐DIO‐hChR2(E123T/T159C)‐EYFP, which expresses a Cre‐dependent Channelrhodopsin (ChR2), into the VTA (**Figure** **7**A). We found that activating VTA‐DA neurons (light‐ON) in *Shank3*‐KO rats during social interaction on the Social versus Empty task resulted in a reduction in investigation time of the social compared to the empty compartment and an overall decrease in investigation of the social stimulus (Figure 7B‐D). When tested on the Social versus Food task, specifically 48 hours after food deprivation, where *Shank3*‐KO rats still showed strong preference to the social stimulus despite food deprivation (Figure 7E,F, light‐OFF), we found that activating VTA‐DA neurons in *Shank3*‐KO rats during social interaction increased their interest in the food over the social stimulus (Figure 7E,F, light‐ON), which was most notable during the third and fourth minutes on interaction (Figure 7G). Collectively, these findings suggest that VTA‐DA neurons hold a pivotal function in governing behavior in response to social and competing rewarding stimuli, and that *Shank3* mutation disrupts this function.

### 2.7 Optogenetic Inhibition of VTA‐GABA Neurons Normalizes the Atypical Behavior in *Shank3*‐*KO* Rats

**Figure 8:**
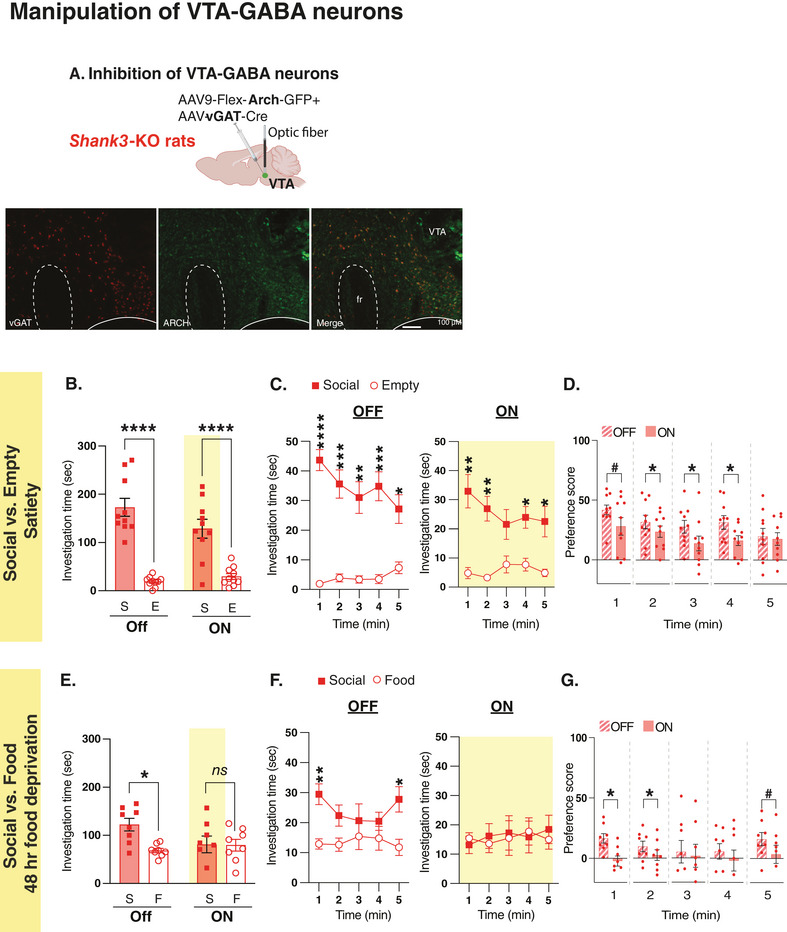
Optogenetic inhibition of VTA‐GABA neurons improves the atypical behavior observed in *Shank3*‐deficient rats. A) A schematic of viral injection and optical fibers placement in the VTA (Top) of *Shank3*‐KO rats and immunohistochemistry staining (bottom) of a sample VTA section showing vGAT expression (left panel, red), ARCH (viral) expression (middle panel, green) and overlap (right panel). 10X magnification; scale bar  = 100 µm. B) Mean total investigation time of the social stimulus or the empty compartment during the non‐ inhibition (OFF) or inhibition (ON) of the VTA‐GABA neurons in *Shank3*‐KO rats (n = 10) when the rats approach the social stimulus at Satiety. C) Mean total investigation time for the social stimulus and the empty compartment averaged in 1‐minute intervals over the 5‐minute test period in *Shank3*‐KO rats. D) Differences in mean investigation time between the social stimulus and the empty compartment for each minute. E‐G) Similar to B‐D, but shows behavioral data during the Social versus Food test at 48 h of food deprivation (KO, n = 8). ****p <.0001, ***p <.001, **p <.01, *p <.05, post hoc tests following the main effect. All error bars represent SEM. Detailed statistical data are provided as a Data file in Table S1 (Supporting Information).

Next, we sought to examine if optogenetic inhibition of VTA‐GABA neurons could also normalize the atypical behavioral phenotype in *Shank3*‐KO rats. To inhibit VTA‐GABA neurons, we injected the rAAV‐hVGAT1‐Cre‐WPRE‐hGH polyA virus along with a Cre‐dependent Arch; AAV9‐FLEX‐Arch‐GFP virus, into the VTA (**Figure** **8**A). We found that inhibition of VTA‐GABA neurons in *Shank3*‐KO rats attenuated the atypical increase in interaction time with the social stimulus, which although was not captured by examining the overall investigation time (Figure 8B), it was clearly detected when examining the behavioral pattern and the preference score across the 5 min of testing (Figure 8C,D). When tested on the Social versus Food task, 48 h after food deprivation where *Shank3*‐KO rats still showed strong preference to the social stimulus despite food deprivation (Figure 8E,F, light OFF), we found that inhibition of VTA‐GABA neurons in *Shank3*‐KO rats during social interaction altered this preference (Figure 8E,F, light ON), mainly during the first and second minutes (Figure 8G). These findings indicate that VTA‐GABA neurons also play a crucial role in regulating behavior during social interaction, and that *Shank3* mutation interferes with this function too.

Our optogenetic experiments thus indicate that manipulating the activity of either DAergic or GABAergic neurons in the VTA enables the restoration of social behavior in *Shank3*‐deficient rats, resembling the behavior observed in WT animals. Altogether, our findings suggest that *Shank3*‐deficient rats exhibit impairment in processing social reward and deriving satisfaction from it, attributable to altered neuronal activity in the VTA

## 3 Discussion

In this study, we employed in vivo recording techniques in *Shank3*‐deficient rats to investigate how *Shank3* mutation affects the real‐time function of the mesoaccumbens pathway during social and non‐social interactions, examining both *Shank3*‐Het and *Shank3*‐KO rats. We included both genotypes to reflect the clinical relevance of the heterozygous condition, which mirrors the loss of one *Shank3* copy observed in humans, and to highlight the significance of studying the homozygous condition, which provides insights into the critical role of Shank3 in specific behavioral aspects and neural circuits. Finally, we used optogenetic tools to assess whether altering neural activity in the mesoaccumbens pathway could influence the rats’ behavior.

By conducting a thorough and impartial examination of behavioral traits, we identified that *Shank3*‐deficient rats displayed an atypical increase in interaction with both newly introduced social and non‐social stimuli when either was presented without any competing stimuli. This atypical behavior was particularly prominent in *Shank3*‐KO rats and more pronounced during interaction with a social stimulus. Furthermore, when *Shank3*‐deficient rats, deprived of food, were exposed to either a juvenile or a toy rat along with food, which held an elevated reward value during the state of food deprivation, an unusually heightened engagement with the social stimulus was observed in both *Shank3*‐Het and KO rats. This phenomenon was notable only in the presence of a juvenile rat stimulus, not a toy rat stimulus. Collectively, these findings highlight the extensive impact of *Shank3* mutations on both social and non‐social interactions in rats. This effect is less pronounced in *Shank3*‐Het rats, likely due to partial compensation by the remaining functional gene copy for the loss of the other, which can mitigate the severity of the phenotype.

Crucially, these effects seem to hinge on the specific context in which they unfold (i.e., presented with one versus two rewarding social or non‐social stimuli) and imply a deficiency in the rat's ability to discern and respond to the positive value of social rewards, resulting in an atypical increase in interaction, that may not necessarily translate to a rewarding experience.

In this context, it is important to emphasize the persistent misconception in the preclinical autism research community that impaired social interactions in ASD invariably lead to a reduction in overall social engagement. Social deficits in ASD and associated neurodevelopmental disorders are highly heterogeneous. Indeed, PMS serves as an exemplar of the unique clinical manifestation of ASD, especially when juxtaposed with idiopathic ASD, which is inherently characterized by its high heterogeneity. This divergence is observable in several key aspects, including the restricted and repetitive behaviors,^[^ ^63^ ^]^ sensory symptoms,^[^ ^64^ ^]^ functional MRI changes in response to social versus non‐social sounds,^[^ ^65^ ^]^ and social deficits, which in contrast to the presentation in idiopathic ASD, do not always manifest as social aversion.^[^ ^66^, ^67^ ^]^ Even within the PMS population, there is considerable variability in the phenotype among affected individuals.^[^ ^37^ ^]^ Notably, phenotypic variability is also observed among *Shank3* models, even within the same species, likely due to differences in targeted gene regions for mutation.^[^ ^68^ ^]^ Additionally, while most *Shank3* mouse models show motor deficits, those are not observed in the rat model.^[^ ^49^ ^]^ Furthermore, the manifestation of the reduced social interaction phenotype is inconsistent among mouse models and is absent in the *Shank3*‐deficient rat model.^[^ ^49^, ^68^ ^]^ Whether these phenotypic variabilities across models are due to a compensatory role of unaffected *Shank3* isoforms or to the different roles of the various isoforms across species remains to be determined and requires further investigation. Another prevailing but mistaken assumption in the field is that behavioral deficits in animal models should perfectly replicate those observed in humans with equivalent genetic alterations. This topic was recently discussed by Silverman et al., who also underscored the importance of the model's construct validity and the utilization of reproducible and validated outcome measures.^[^ ^69^ ^]^ This approach, as opposed to trying to artificially force the model into a human‐like phenotype, optimally advances our goal of deepening our understanding of the mechanistic effects of mutations associated with neurodevelopmental disorders.

The increased social interaction we observed in *Shank3*‐deficient rats and their inability to adjust behavior according to the rewarding value of the presented stimulus, prompted us to test the hypothesis that deficits in the mesoaccumbens reward pathway could be a contributing factor. The role of VTA‐DA neurons in learning and motivation, as well as in reward processing has been well‐established in both clinical and preclinical studies.^[^ ^10^, ^11^, ^16^, ^17^, ^18^, ^19^ ^]^ Specifically, studies in mice have highlighted the critical involvement of VTA‐DA neurons in the processing of social rewards, with increased activity noted during social interactions.^[^ ^46^, ^47^, ^57^, ^59^ ^]^ Consistent with these observations, our findings demonstrated, for the first time, increased activity in VTA‐DA neurons also in rats (WT rats) when they engaged with a social stimulus. However, this pattern of activity was not observed in *Shank3*‐deficient rats, despite their increased interaction with the social stimulus. Additionally, when compared to their WT littermates, *Shank3*‐deficient rats exhibited increased VTA‐GABA activity during social interaction. These deficiencies are in alignment with earlier findings from in vitro and in vivo single unit recordings conducted in mice, which demonstrated that reducing Shank3 levels in the VTA during early postnatal development led to decreased activity in DA neurons and increased activity in GABA neurons, which was linked to an impaired maturation of excitatory synapses on these neurons.^[^ ^47^ ^]^ The question of whether the lack of maturation in excitatory synapses on VTA neurons accounts for the impaired VTA neural activity during social interaction in the rat model remains a subject for future investigations. In this context, it is important to emphasize that the activity of VTA‐DA and VTA‐GABA neurons is modulated not only by excitatory but also inhibitory inputs from various brain regions, including inputs from the bed nucleus of the stria terminalis (BNST), lateral hypothalamus (LH), NAc, and medial preoptic area (MPOA).^[^ ^17^, ^70^, ^71^, ^72^ ^]^ Activation of these inputs was demonstrated to induce a pro‐reward behavior, increased reward consumption, and/or reduced anxiety.^[^ ^73^ ^]^ In the realm of social reward, activation of the LH–VTA GABA pathway has been demonstrated to enhances social interaction. This occurs via suppression of VTA‐GABA neurons and facilitation of DA release in the nucleus accumbens.^[^ ^74^ ^]^ A recent study in rats has shown that inhibition of projections from the central nucleus of the amygdala onto VTA‐GABA neurons disrupts the maintenance, but not the initiation, of social interaction, potentially due to disinhibition of DA neurons in the VTA.^[^ ^75^ ^]^ These findings together propose a model in which local VTA‐GABA circuits shape social behavior. They also imply that the influence of a deficiency in Shank3 on VTA‐DA neurons may occur indirectly through its impact on the intricate nature of the VTA‐GABA population. Therefore, future investigations should further explore the impact of *Shank3*‐deficiency on VTA local circuits to advance our comprehension of the pathophysiology related to *Shank3*‐deficiencies. Additionally, considering the diverse nature of VTA neural populations and the intricate network of inputs and outputs within the VTA,^[^ ^17^, ^70^, ^71^, ^72^ ^]^ as well as their distinct roles in various behaviors, including feeding, learning and motivation, and social behavior,^[^ ^73^ ^]^ it is imperative for future studies to dissect the specific influence of *Shank3* in particular VTA pathways, encompassing both social and non‐social behaviors. An initial step towards addressing this broader question could involve mapping the expression of *Shank3* in subpopulations of neurons and specific VTA inputs and outputs.

Extensive research has established the pivotal role of VTA‐DA to NAc projecting neurons and DA release in the NAc in motivated behaviors, reward processing, and social behavior.^[^ ^10^, ^11^, ^16^, ^17^, ^18^, ^19^ ^]^ Moreover, VTA‐GABA neurons were shown to inhibit the activation of VTA‐DA neurons potentially perturbing DA release in the NAc.^[^ ^76^ ^]^ Pertaining to social behavior, studies in rats have demonstrated that variations in DA levels in both the dorsal and ventral striatum correspond with episodes of social interaction.^[^ ^77^, ^78^ ^]^ Moreover, a prior study in mice has elucidated that social interaction leads to heightened activity, particularly in VTA‐DA neurons projecting to the NAc.^[^ ^57^ ^]^ These observations align with our findings in WT rats, illustrating an increase in DA release in the NAc during social interaction. This increase likely plays a crucial role in encoding the rewarding value of social interactions, leading to a gradual decrease in motivation for further social engagement once that reward is registered and satisfaction is achieved. Our findings in *Shank3*‐KO rats, which highlight that reduced DA release in the NAc and compromised activity in both VTA‐DA and VTA‐GABA neurons are linked to an atypical increase in social interaction, suggest that these rats experience a deficiency in processing the rewarding value of social interaction. As a consequence of this deficiency, there is reduced satisfaction from social interactions, prompting them to pursue more of these interactions without properly adapting their behavior to other rewarding stimuli in their surroundings.

Overall, our findings demonstrate that Shank3 plays a critical role in modulating the activity of VTA neurons and the VAT‐NAc pathway, which plays pivotal role during reward processing of social stimuli. In support of this argument, our optogenetic interventions provided evidence of a causal relationship between the observed deficits in neural activity and the atypical social interactions in the *Shank3* rat model. Additionally, our discovery that this atypical interaction could be ameliorated by augmenting the activity of VTA‐DA neurons or mitigating the excessive activity of VTA‐GABA neurons in *Shank3*‐deficient rats during adulthood implies that there may be opportunities to intervene in the VTA pathway and potentially improve social behavioral deficits associated with *Shank3‐*deficiencies.

## 4 Experimental Section

### Animals

We used adult (8‐16 weeks old) male *Shank3*‐Heterozygous (Shank3‐HET), *Shank3*‐homozygous/knockout (*Shank3*‐KO) and their wildtype (WT) littermate rats. *Shank3*‐deficient rats were created using zinc‐finger nucleases on an outbred Sprague‐Dawley background, introducing a 68 bp deletion in exon six. This deletion leads to a premature stop codon, disrupting the largest isoform, Shank3a, as previously reported.^[^ ^49^ ^]^ Rats were kept under veterinary supervision in a 12 h reverse light/dark cycle at 22 ± 2 °C. Animals were pair‐caged with food and water available ad libitum, except during a 48 h period of food deprivation on the Social versus Food task when they had access to water but not food. Experiments were conducted during the light phase cycle. All animal procedures were approved by the Institutional Animal Care and Use Committees at the Icahn School of Medicine at Mount Sinai and the University of Haifa.

### Stereotaxic Surgeries for Viral Injections

8‐week‐old animals were anesthetized with 3% isoflurane for induction. Isoflurane was then maintained at 1.5‐2.5% with 2% oxygen, using a tabletop vaporizer and a non‐breathing circuit. The surgical area was shaved and sterilized before a vertical incision was made along the midline of the skull. After clearing the connective tissue, bregma and lambda were identified, the region of injection was marked, and a small burr hole (50 µm) was drilled. The rats were injected unilaterally (for the fiber photometry experiments) or bilaterally (for the optogenetic experiments) with the appropriate viruses. Viruses were loaded into a 10 µl 33G NanoFil syringe (World Precision Instruments, Sarasota, FL, USA) and 0.350 µL of the virus was injected into the VTA (A‐P ‐5.6 mm, M‐L 0.9 mm, D‐V 8.0 mm) at a rate of 0.1 µL min^−1^. Following injection, the syringe was left in place for 5 min and withdrawn at a rate of 0.2 mm min^−1^. Immediately after, a fiber optic cannula (400 µm 0.39 NA, Cat. CFM14L10, Thor Labs, Newton, New Jersey) was implanted in the same coordinates for the fiber photometry or bilaterally with a 10° angle for the optogenetic experiments. The incision wound was closed using sutures (Ethilon Suture 5‐0, Henry Schein, Melville, NY, USA). Animals received intraoperative subcutaneous fluids (Lactated Ringer Solution, Thermo Fisher Scientific, Waltham, MA, USA) and buprenorphine (0.5 mg kg^−1^) for analgesia. Additional analgesia was administered subcutaneously every 12 h for 72 h post‐operatively.

### Viral Vectors

All the viruses used in this study have been previously employed by several other groups.^[^ ^62^, ^79^, ^80^, ^81^, ^82^, ^83^, ^84^ ^]^ To record from VTA‐DA neurons, we used a combination of AAV9.rTH.PI.Cre.SV40^[^ ^81^, ^84^ ^]^ (Addgene 107 788) and AAV9‐CAG‐FLEX‐GCaMP6m.WPRE.SV40^[^ ^79^ ^]^ (Addgene 100 841). We validated the high specificity of this viral combination in targeting DA neurons by evaluating the percentage of GCaMP6m‐positive neurons that co‐express TH, a marker for DA neurons. Our analysis revealed that 82.8% of GCaMP6m‐expressing neurons were also TH‐positive (Figure S1A, Supporting Information). To record from VTA‐GABA neurons we used a combination of rAAV‐hVGAT1‐Cre‐WPRE‐hGH polyA^[^ ^62^ ^]^ (biohippo, Cat no# PT‐0346) and AAV9‐CAG‐FLEX‐GCaMP6m^[^ ^79^ ^]^ (Addgene 100 841). We also validated the high specificity of this viral combination in targeting GABA neurons by evaluating the percentage of GCaMP6m‐positive neurons that co‐express vGAT, a marker for GABA neurons. Our analysis revealed that 89.6% of GCaMP6m‐expressing neurons were also vGAT‐positive (Figure S1B, Supporting Information). To record from DA release in the NAc, we used a GRAB sensor AAV9‐hSyn‐DA2m (DA4.4)^[^ ^83^ ^]^ (WZ Biosciences). To activate VTA‐DA neurons in *Shank3*‐deficient rats, we used a combination of AAV9.rTH.PI.Cre.SV40^[^ ^81^, ^84^ ^]^ (Addgene 107 788) and AAV9‐Ef1a‐DIO‐hChR2(E123T/T159C)‐EYFP^[^ ^82^ ^]^ (Addgene 35 509). To inhibit VTA‐GABA neurons in *Shank3* ^−^deficient rats, we used a combination of rAAV‐hVGAT1‐Cre‐WPRE‐hGH polyA^[^ ^62^ ^]^ (biohippo) and AAV9‐FLEX‐Arch‐GFP (Addgene 22 222).^[^ ^80^ ^]^

### Behavioral Assays

*Social/Object Versus Empty Task*: Rats were habituated to a testing arena containing two empty compartments on opposite corners of the arena for 15 min, as previously described.^[^ ^54^ ^]^ Notably, the arena included two other empty corners that the rats use for non‐investigative activities, serving as neutral spaces. Unlike the three‐chamber task, our testing arena lacks a middle chamber, which typically encourages non‐investigative behavior. This design was intentional to promote more interaction with the stimuli.^[^ ^54^, ^85^ ^]^ A social stimulus (novel male Juvenile, 4 weeks old) or a moving object (toy rat) was then placed into one compartment with the opposite compartment left empty (counterbalanced). Compartments had a wire mesh window which allowed for the exchange of visual, auditory, and olfactory information, with very limited physical touch. The subject rat was then allowed to interact for 5 min.

*Social/Object Versus Food Task at Satiety and after 48 H of Food Deprivation*: Rats were habituated to a testing arena containing two empty compartments on opposite corners of the box for 15 min, as previously described.^[^ ^54^ ^]^ A social stimulus (novel male juvenile, 4 weeks old) or a moving object (toy rat) was then placed into one compartment and food pellets were placed at the opposite compartment (counterbalanced). The subject rat was then allowed to explore the arena for 5 min. The task was repeated after 48 h of food deprivation with new juveniles and both compartments on the opposite corners randomly rearranged to avoid habituation and learning.

*Food Consumption Task at 48 H of Food Deprivation*: After 48 h of food deprivation, rats were habituated to a testing arena containing two empty compartments on opposite corners of the box for 15 min. Three tests were carried out:

1. Food pellets were placed in the middle of the open field. The subject rat was allowed to explore and consume the pellets for 5 min.
2. A social stimulus (novel male juvenile, 4 weeks old) was placed into one compartment with the opposite compartment left empty, and food pellets were placed in the middle of the open field. The subject rat was then allowed to explore and consume the pellets for 5 min.
3. A moving object (toy rat) was placed into one compartment with the opposite compartment left empty, and food pellets were placed in the middle of the open field. The subject rat was then allowed to explore and consume the pellets for 5 min.

The amount of grams consumed was determined by measuring the initial weight of the food pellets and then subtracting the weight of the remaining pellets after testing.

*Fiber Photometry Recording and Analysis*: Calcium signal recording was conducted using fiber photometry (RZ10x; Tucker‐Davis Technologies, FL, USA). The 465 and 405 nm LEDs were driven at 300 mA and 100 mA respectively, with the power at the tip of the fiber optic cannula determined to be at 80 uW. A USB camera (C615 portable HD webcam, Logitech) was placed at the top of the acoustic chamber and connected to a computer for video recording of the subject rat behavior (≈15 frames per second). The video clip recorded by the USB camera was synchronized to the calcium signal as described in the TDT manual (https://www.tdt.com/docs/synapse/hardware/video‐processors/#rv2‐video‐tracker). TDT Synapse software (TDT) was used for recording the signal channel (excitation 470 nm), the isobestic control channel (405 nm), and the digital channel receiving the camera strobes. Three weeks following viral injection and fiber implantation, rat subjects were first habituated to the testing arena for 15 min. Calcium signals were then recorded for 5 min before and 5 min after introducing the stimulus/stimuli, by connecting a fiber optic patch cord (400 µm, 0.48 NA, Doric lenses, Quebec, Canada) to the fiber optic cannula.

Calcium signal data was analyzed following a previously published pipeline, using a custom‐written MATLAB Script.^[^ ^86^ ^]^ First, we fitted the 405 channels onto 465 channels to detrend signal bleaching and any movement artifacts, according to the manufacturer's protocol (https://github.com/tjd2002/tjd‐shared‐code/blob/master/matlab/photometry/FP_normalize.m). Next, the signal was aligned to the video recording using the timestamps recorded by the digital port of the RZ10× system. Calcium signal was aligned to each event and normalized using *z* score (0.1 s bins; zdF/F), where the 2‐s pre‐event period served as the baseline. The duration of the pre‐event period was determined to be 2 s since our analysis revealed that the majority of transitions between the two stimuli took 2 s (Figure S2, Supporting Information). Heatmaps were created to depict the fluctuation in fluorescence signals (zdF/F) during the period ranging from 2 s before to 5 s after each interaction bout. To validate the presence of a signal in fiber photometry recordings, we ensured a stable baseline before introducing stimuli and assessed the signal‐to‐noise ratio to confirm that the detected signal was significantly above background noise. All rats included in our analysis exhibited a positive response to either food or both social and food stimuli, thereby ruling out the possibility of no response due to technical issues. To confirm that the bouts of interaction represent genuine social engagement rather than mere immobility or other behaviors, we randomly selected six rats per genotype and manually scored their active interaction, immobility, rearing, and grooming behavior during the social versus food task at satiety while blinded to their genotype. Our findings revealed that non‐interaction behaviors were rare, confirming that the FP signals are primarily associated with active interaction (Figure S3, Supporting Information). At the end of the experiments, all rats were perfused, and their brains were collected and processed for immunohistochemistry (IHC) to verify the correct positioning of the fiber and the presence of viral expression. Rats that did not meet these criteria were excluded from the study. It is important to note that certain rats never engaged with one of the stimuli, resulting in a lack of photometry data. Consequently, these rats were not included in the heatmap, causing variations in the number of rows (representing rats) between the two stimuli. Signal changes were quantified for relevant time intervals as the corresponding areas under the curve for the averaged z‐scores, which was calculated using GraphPad software (GraphPad Prism, San Diego, CA, USA) using the ‘area under the curve’ function.

### Optogenetics and Behavior

Optogenetic activation or inhibition experiments were conducted three weeks following viral injections and fiber implantations. Rats were first habituated to the testing arena for 15 min. Then a splitter fiber optic patch cord (400 µm, 0.37 NA, Doric lenses, Quebec, Canada) was connected to the implanted bilateral fibers (CFM14L10, Thorlabs Inc.) through sleeves (F210‐3012, Doric lenses) to deliver blue (activation, 465 nm, 5 Mw, 5 Hz for the DA neurons with a 20 ms pulse width and 10 Hz for the vGAT neurons with a 10 ms pulse width) or yellow (inhibition, continuous, 560 nm, 5 mW) light (Doric lenses). The stimuli were then introduced, and the behavior was recorded for 5 min. The light used to stimulate neural activity was manually delivered only during periods of social interaction (light ON), while the light used to inhibit neural activity was delivered throughout the entire testing session. Rats were randomly assigned, with half starting the first round of testing with the lights ON and half with the lights OFF. Then, on an independent day for the second round of testing, those that had the lights ON during the first round switched to having the lights OFF, and vice versa.

### Behavioral Analysis

All behaviors were scored and quantified using TrackRodent, an open‐source Matlab based automated tracking system (https://github.com/shainetser/TrackRodent) that uses a body‐based algorithm (*WhiteRatBodyBased15_7_15* in the TrackRodent interface)^[^ ^54^, ^85^, ^87^ ^]^ Investigation time was automatically assessed based on active contact between the subject's body and the stimulus's chamber using the TrackRodents software.^[^ ^85^ ^]^ Bout duration was defined as a continuous contact between the subject's body and stimulus's chamber with no gaps longer than 0.5s

The behavioral traces and heat‐maps were obtained using a MATLAB custom‐made code (https://zenodo.org/records/10222543). Videos were de‐identified in order to keep the experimenter blinded to the treatment groups while setting up the analysis. We pooled together all behavioral data for each task, which allowed us to effectively increase the sample size. This was made possible because the different cohorts of rats (each administered with different virus combinations) were all assessed on identical tasks. This enhanced the statistical power of our findings and allowed us to gain a more comprehensive understanding of the behavioral characteristics. However, to maintain clarity and transparency, we present each cohort's data separately in the Extended Data, and statistical analysis for each cohort is listed in the Table S1 (Supporting Information).

*Investigation Time*: Behavioral analysis was done after correcting the raw behavioral data by considering any gap of < 0.5 s in investigation of a given stimulus as part of the same investigation bout. Investigation time was calculated in 1‐minute bin across all tests.

*Investigation and Number of Bouts*: Given that we have previously reported that longer bouts are associated with social interaction, rather than general exploration,^[^ ^54^ ^]^ we categorized the different investigation bouts according to their length (< 6 s, 6 s, and >19 s), and calculated investigation time and number of each for each duration category, as done before.^[^ ^54^ ^]^

*Transitions*: A transition between stimuli was defined as the time point when investigation of a new stimulus (relative to the other stimulus) started. The mean rate of transitions was calculated at 1‐min bins.

*Interval Duration*: We evaluated intervals between bouts in which the animal shifted from one stimulus to the other. We counted the number of intervals according to their length and calculated their percentage for each interval duration.

### Histology

After the completion of behavioral testing, rats were deeply anesthetized with 4% isoflurane in an induction chamber. Once reaching a surgical plane of anesthesia, rats were perfused transcardially at a rate of 40 mL min^−1^ with 0.9% NaCl, followed by ice‐cold 4% paraformaldehyde (PFA) fixative in 0.1 M phosphate buffer saline (PBS) at pH 7.4. Brains were extracted, post‐fixed in 4% PFA for 12 h at 4 °C, and cryoprotected by immersion in a 15% sucrose solution in 0.1 M PBS for 24 h at 4 °C. Prior to sectioning, brains were flash frozen in isopentane and stored at −20 °C. Brains were sectioned with a cryostat (Leica CM 1860 Leica Biosytems, Buffalo Grove, IL, USA) and a series of 30 µm thick coronal sections of the VTA or NAc region, collected in a cryoprotective solution (1:1:2 glycerol/ethylene glycol/phosphate buffer saline) and stored at 4 °C.

### Immunofluorescent Staining

Brain sections were rinsed in 1X PBS with 0.05% Triton X‐100 (PBS‐T), then incubated in a primary antibody anti‐tyrosine hydroxylase (1:2000, mouse, monoclonal, MAB318, EMD Millipore) and/or anti‐GFP (1:2000, chicken, A10262, invitrogen) overnight at 4 °C in 10% nonfat dry milk and 1% bovine serum albumin (BSA) in 0.03% Triton X‐100 in 1xPBS. The following day, sections were washed with 1X PBS with 0.05% Triton X‐100 (PBS‐T) and incubated in secondary antibody (Cy3, Donkey anti‐mouse, code: 711‐165‐152, and Alexa Fluor 488, Donkey anti‐chicken, code:703‐545‐155, Jackson Immuno Research) (1:1000 in 0.5% Triton X‐100 in PBS) for 2 h at room temperature. Sections were then rinsed and mounted with VECTASHIELD Antifade Mounting Medium with DAPI (cat. no. H‐1200, Vector Labs, Burlingame, CA, USA).

### RNAscope

Rat *vgat1 (slc32a1)* probe was purchased from ACDBio. After perfusion, extraction, post‐fixation, and sucrose immersion, brains were flash frozen in a slurry of isopentane and dry ice. Tissue was sectioned at 20 µm, mounted on glass slides (SuperFrost Plus Microscope Slides, Fisher Scientific, USA), and frozen at −80 °C until the day of experiment. RNAscope was performed following the manufacturer's protocol (RNAscope Multiplex Fluorescent Reagent Kit, ACDBio, USA). Briefly, tissue sections were thawed at RT for 10 min, fixed with 4% PFA for 15 min at 4 °C, and dehydrated with varying ethanol concentrations. They were then incubated in H_2_O_2_ for 10 min and the *vgat1* probe added and incubated for 2 h in a 40 °C oven (HybEZ II Hybridization System, ACDBio, USA). This was followed by an amplification step and then incubation with opal dye (Akoya Biosciences, USA) 570 to visualize the RNA transcripts. Immunohistochemical staining for GFP was performed following the RNAscope experiment with a blocking step (1 h, RT) with donkey serum and incubation of tissue with anti‐GFP (1:1000, chicken, A10262, invitrogen) overnight at 4 °C followed by incubation with Donkey‐anti‐Chicken 488 (2h, RT).

### Image Acquisition and Processing

Immunofluorescent staining and fluorescent in situ hybridization (RNAscope) were imaged on a Zeiss AxioImager Z2M with ApoTome.2 at 10x magnification at the Microscopy and Advanced Bioimaging CoRE at the Icahn School of Medicine at Mount Sinai.

### Statistical Analysis

All statistical analyses were performed using GraphPad prism 9.0 software (GraphPad Prism, San Diego, CA, USA). Detailed information about statistical tests is provided in each figure legend and in the Table S1 (Supporting Information). Image graphics were created using BioRender.com and Adobe Illustrator.

## Conflict of Interest

The authors declare no conflict of interest.

## Author Contributions

M.B. and H.H.N. conceptualized and designed the study. M.B. performed all experiments and acquired all imaging data. K.T.R. helped in the behavioral analysis and contributed to the conceptualization of the study and the manuscript preparation. S.N. and S.W. provided support on the open‐source behavioral analysis software and manuscript preparation. M.B. and H.H.N. interpreted the data, prepared figures, and wrote the manuscript. All authors read and approved the final manuscript.

## Supporting information



Supporting Information

Supporting Information

## Data Availability

The data that support the findings of this study are available from the corresponding author upon reasonable request.
