# Supplementary material for: Altered Neural Activity in the Mesoaccumbens Pathway Underlies Impaired Social Reward Processing in Shank3‐Deficient Rats
Source: Adv Sci (Weinh). 2025 Mar 14;12(17):2414813. doi: 10.1002/advs.202414813 (PMC12061274; doi:10.1002/advs.202414813)
Supplement: Supplementary file 1 — Supporting Information [file ADVS-12-2414813-s001.docx]

Extended Data Figure 1.

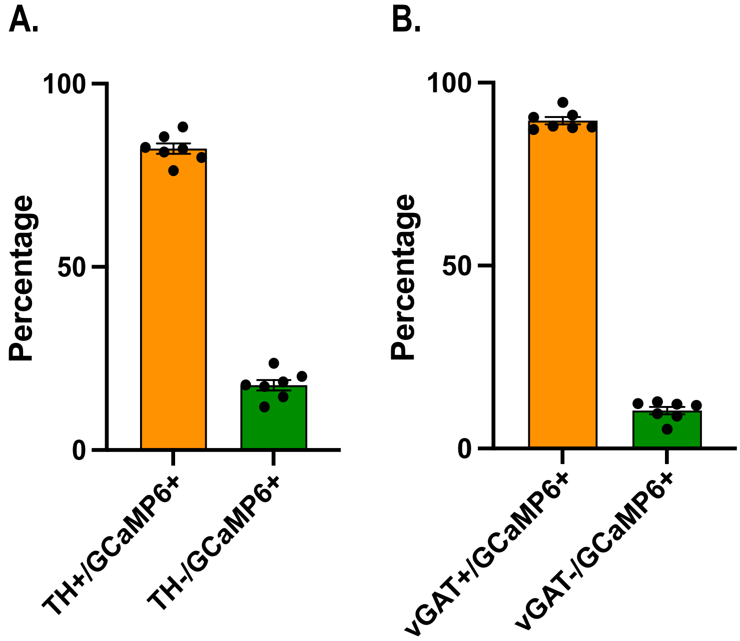

Extended Data Figure 1. A. Percentage of neurons co-expressing TH-Cre and DIO-GCaMP6 in the VTA ($GCaMP6^+/TH^+$) compared to neurons expressing GCaMP6 but not TH ($GCaMP6^+$/ TH^-) (n=7 rats). **B.** Percentage of neurons co-expressing vGAT-Cre and DIO-GCaMP6 in the VTA ($GCaMP6^+/vGAT^+$) compared to neurons expressing GCaMP6 but not vGAT ($GCaMP6^+/vGAT^-$) (n=7 rats).

Extended Data Figure 2.

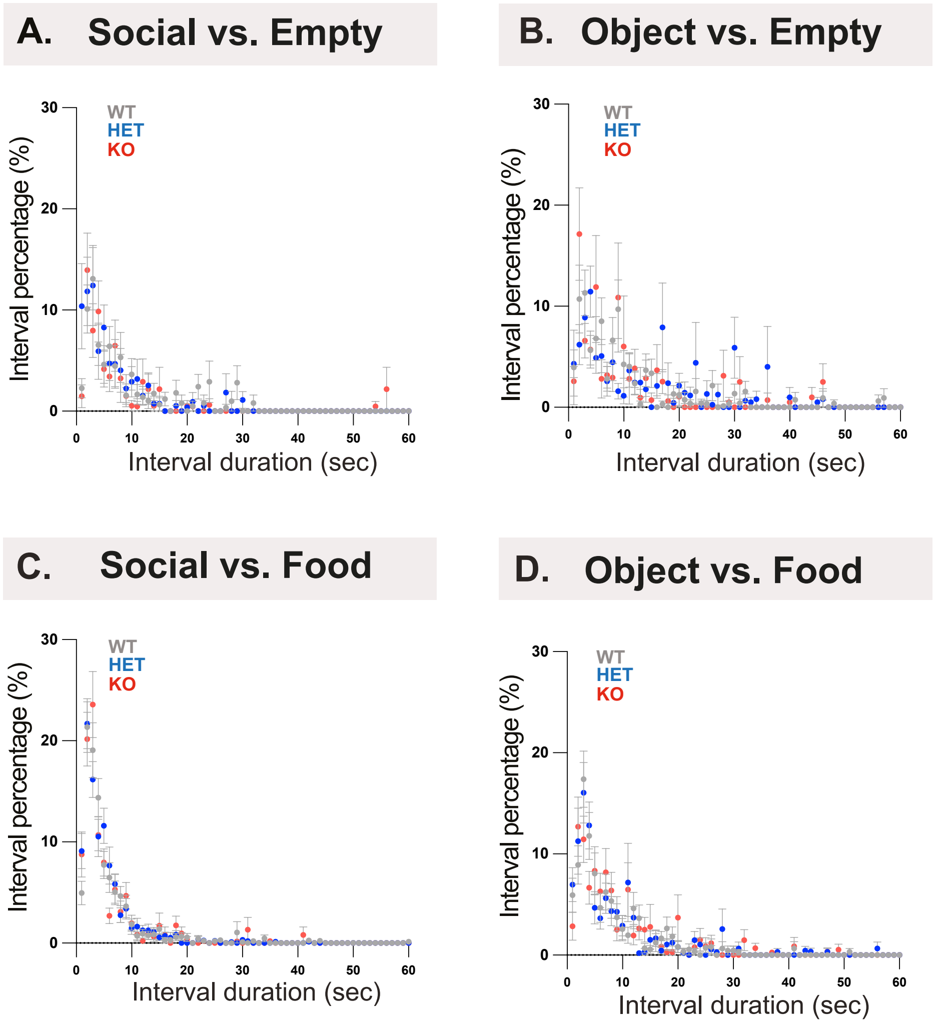

Extended Data Figure 2. A. Mean percentage of each interval length (using 10 sec bins) during the 5-minutes of testing on the Social vs. Empty task at Satiety. Intervals duration represent the time it takes an animal to transition from one compartment to another. WT, n=25; HET, n=26; KO, n=23. **B.** Similar to A, but shows behavior during the Object vs. Empty task at Satiety. WT, n=29; HET, n=30; KO, n=27. **C** and **D**. Mean percentage of each interval length (using 10 sec bins) during the 5-minutes of testing on the Social vs. Food task (C, WT, n=47; HET, n=47; KO, n=41) or Object vs Food task (D, WT, n=25; HET, n=26; KO, n=23) at Satiety. Intervals duration represent the time it takes an animal to transition from one compartment to another. Detailed statistical data are provided as a Data file in Extended Data, Table 1.

Extended Data Figure 3.

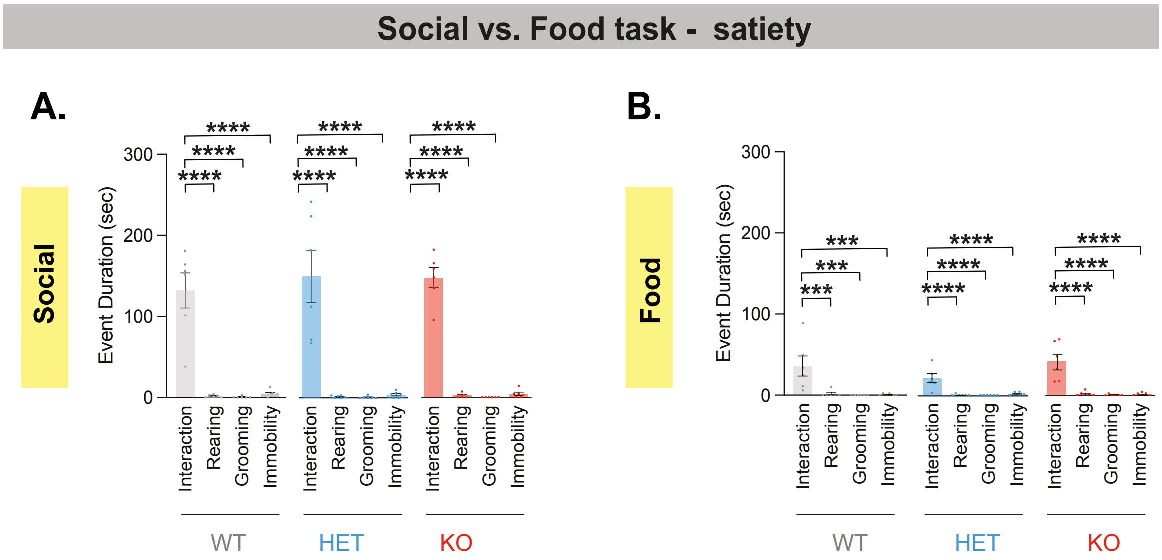

Extended Data Figure 3. A. Mean total duration of interaction, grooming, rearing, and immobility events during social exploration in the Social vs. Food task at Satiety. **B.** Similar to A, but displaying behavioral data during food exploration. WT, n=6; HET, n=6; KO, n=6. *****p*<.0001, ***p*<.01, **p*<.05, based on post hoc tests following the main effect. Error bars represent SEM. Detailed statistical data are provided as a Data file in Extended Data, Table 1.

Extended Data Figure 4.

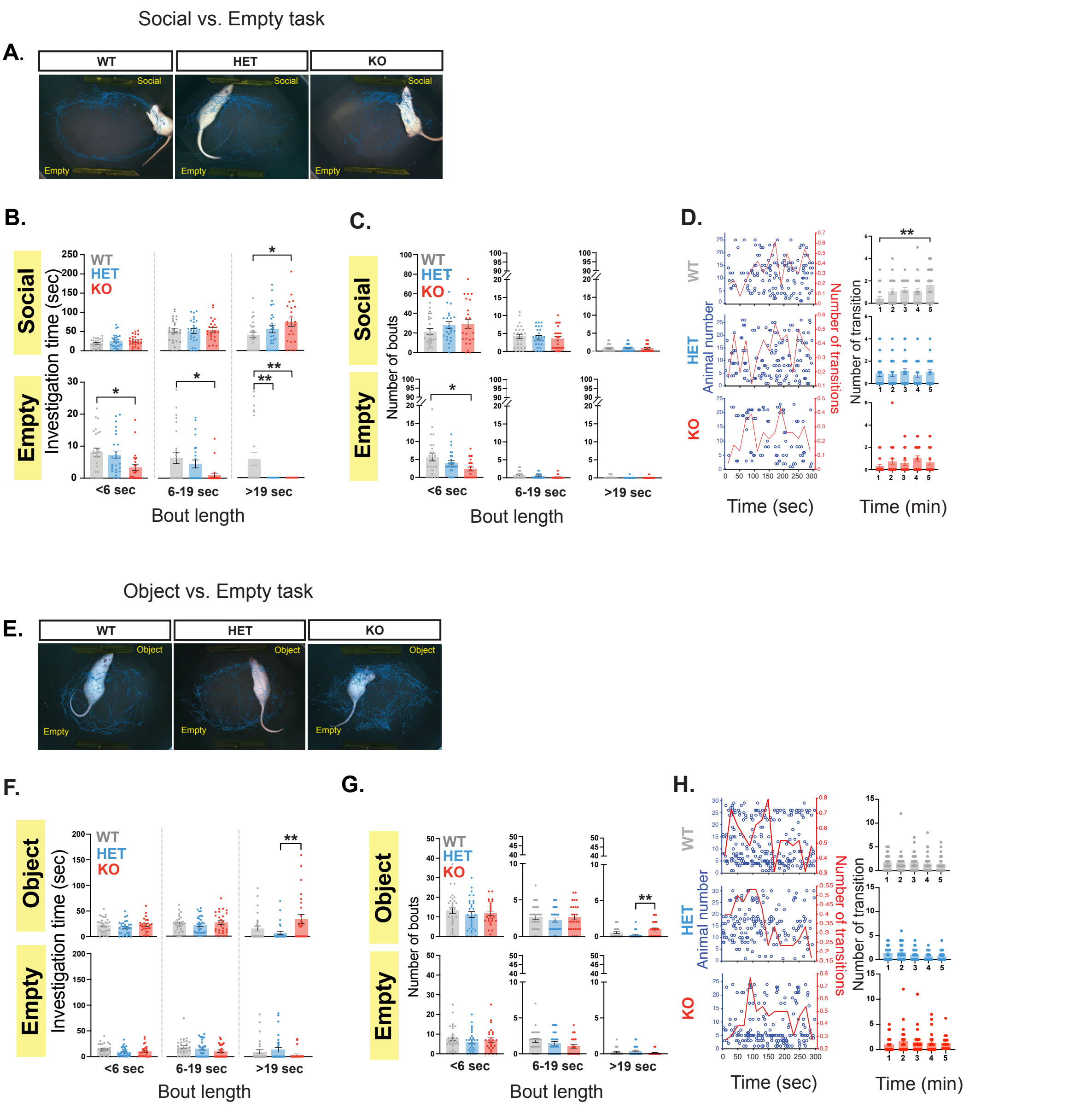

Extended Data Figure 4. A. A representative trace (from one rat per genotype) during the Social vs. Empty task at Satiety. **B.** Mean total duration of short investigation bouts (<6 sec), medium length bouts (6-19 sec), and long investigation bouts (>19 sec) during the Social vs. Empty task during investigation of the social-containing (top) or empty compartment (bottom). **C.** Mean combined number of investigation bouts (<6 sec), medium length bouts (6-19 sec), and long investigation bouts (>19 sec) during the Social vs. Empty task during investigation of the social-containing (top) or empty compartment (bottom). **D.** Left, Transitions between the two

compartments across time during the Social vs. Empty task. Each punctum denotes the beginning of investigation of a new stimulus, and each row represents a single subject. The mean rate (using 20-s bins) is denoted by the red line (right red y-axis). Right, Mean pooled number of transitions between the two compartments (social or empty) over the 5-minute test period. WT, n=25; HET, n=26; KO, n=23. **E-H.** Similar to A-D, but shows behavior during the Object vs. Empty task at Satiety. WT, n=29; HET, n=30; KO, n=26. $^{**}p<.01$, $^{*}p<.05$, post hoc tests following the main effect. All error bars represent SEM. Detailed statistical data are provided as a Data file in Extended Data, Table 1.

Extended Data Figure 5.

TH-Cre Cohort

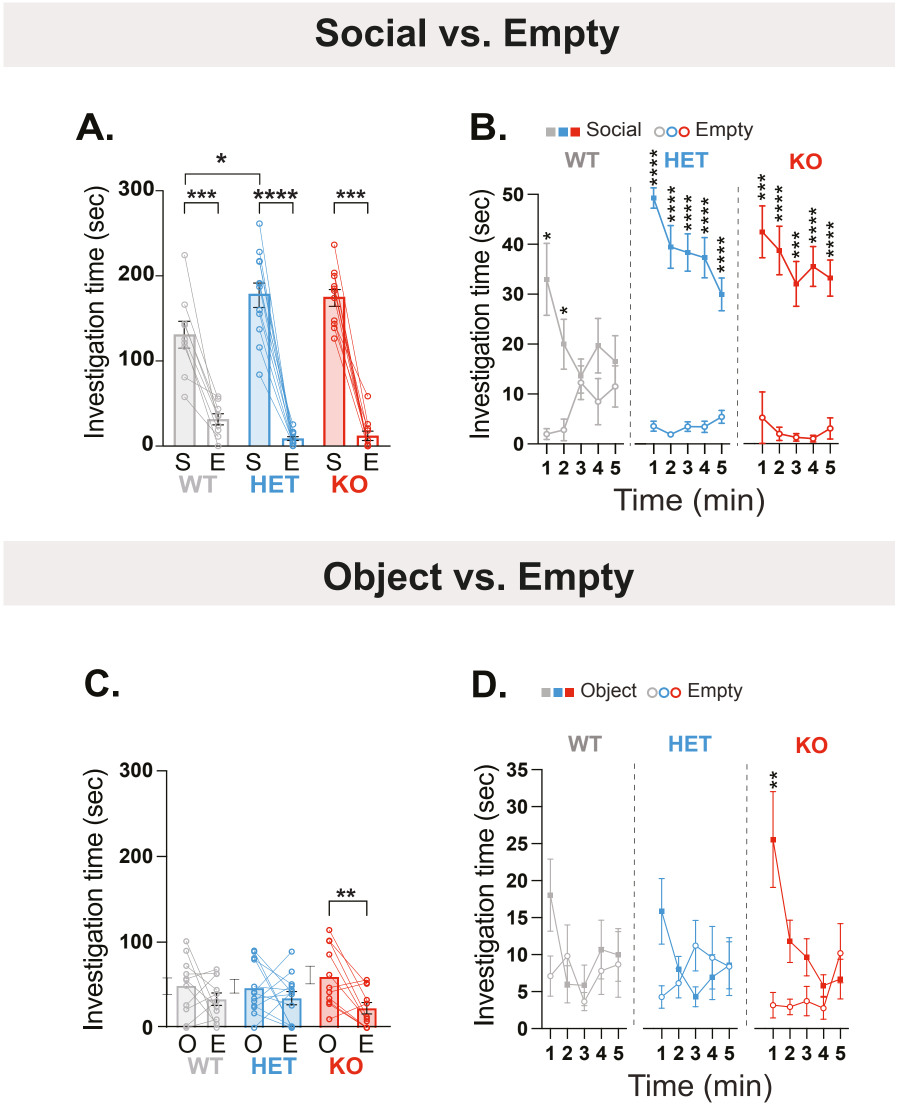

Extended Data Figure 5. A. Mean total investigation time of the social stimulus or the empty compartment for the cohort of rats injected with TH-Cre and DIO-GCamp6 viruses, during testing on the Social vs. Empty task at Satiety. **B.** Mean total investigation time for the social stimulus and the empty compartment averaged in 1-minute intervals over the 5-minute test period. WT, n=9; HET, n=12; KO, n=11. **C and D.** Similar to A and B, but shows behavior during the Object vs. Empty task at Satiety. WT, n=11; HET, n=13; KO, n=11. ****p<.0001, ***p<.001, **p<.01, *p<.05,

post hoc tests following the main effect. All error bars represent SEM. Detailed statistical data are provided as a Data file in Extended Data, Table 1.

Extended Data Figure 6.

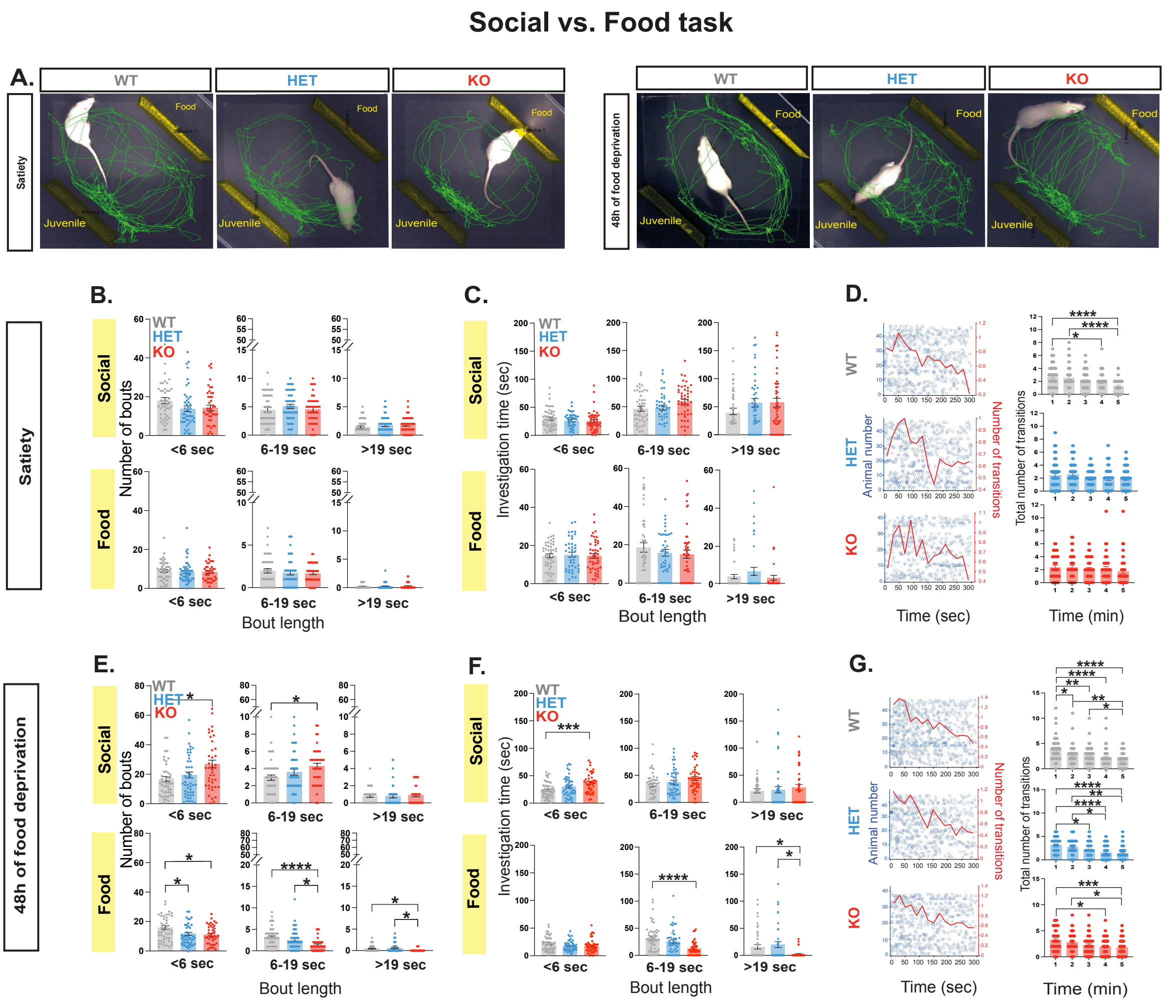

Extended Data Figure 6. A. A representative trace (from one rat per genotype) during the Social vs. Food task, at satiety (left) and after 48 hours of food deprivation (right). **B.** Mean combined number of investigation bouts (<6 sec), medium length bouts (6-19 sec), and long investigation bouts (>19 sec) during the Social vs. Food task at satiety during investigation of the social-containing (top) or food compartment (bottom).**C.** Mean combined duration of short investigation bouts (<6 sec), medium length bouts (6-19 sec), and long investigation bouts (>19 sec) during the Social vs. Food task during investigation of the social-containing (top) or food compartment (bottom). **D**. Left, Transitions between the two compartments across time during the Social vs. Food task. Each punctum denotes the beginning of investigation of a new stimulus, and each row represents a single subject. The mean rate (using 20-s bins) is denoted by the red

line (right red y-axis). Right, Mean pooled number of transitions between the two compartments (social or food) over the 5-minute test period. **E-G**. Similar to B-D, but shows behavior during the Social vs Food task after 48 hours of food deprivation. WT, n=47; HET, n=47; KO, n=41. ****$p<.0001$, *$p<.05$, post hoc tests following the main effect. All error bars represent SEM. Detailed statistical data are provided as a Data file in Extended Data, Table 1.

Extended Data Figure 7.

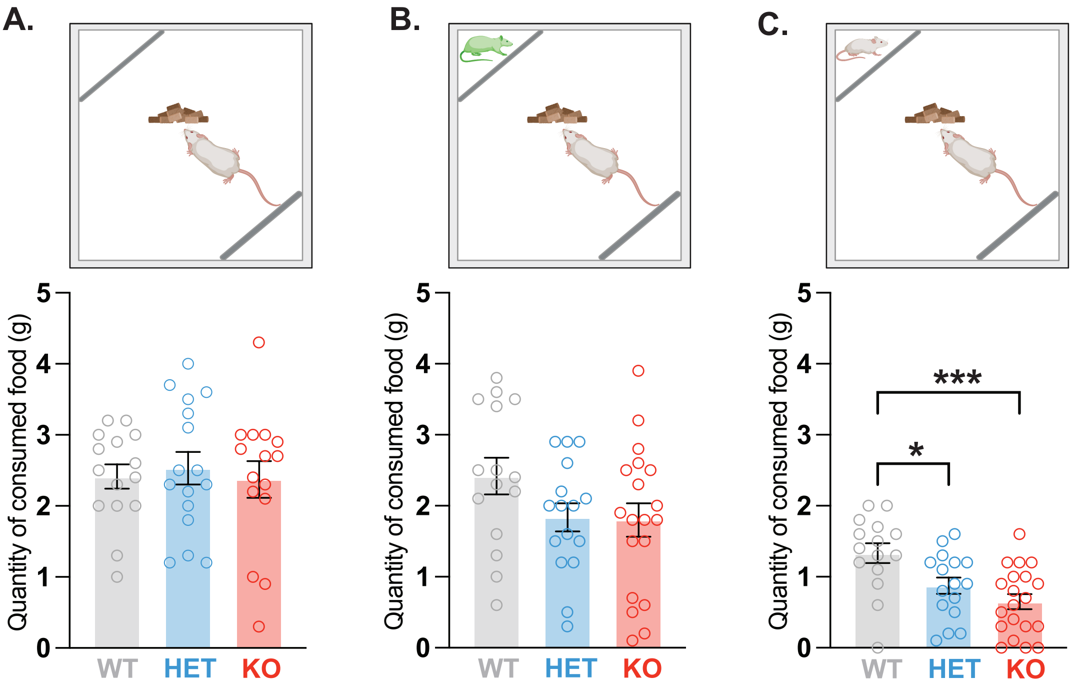

Extended Data Figure 7. A. Outline for the Free Food Intake Test (Top) after 48 hours of food deprivation. Graph depicting the average food intake during the 5-minute test period where no stimulus is presented (Bottom). **B.** Similar to A, but shows food intake during the Object vs. Empty task at 48 hours of food deprivation. **C.** Similar to A, but shows food intake during the Social vs. Empty task at 48 hours of food deprivation. WT, n=15; HET, n=16; KO, n=15. All error bars represent SEM. ***p<.001, *p<.05, post hoc tests following the main effect. Detailed statistical data are provided as a Data file in Extended Data, Table 1.

Extended Data Figure 8.

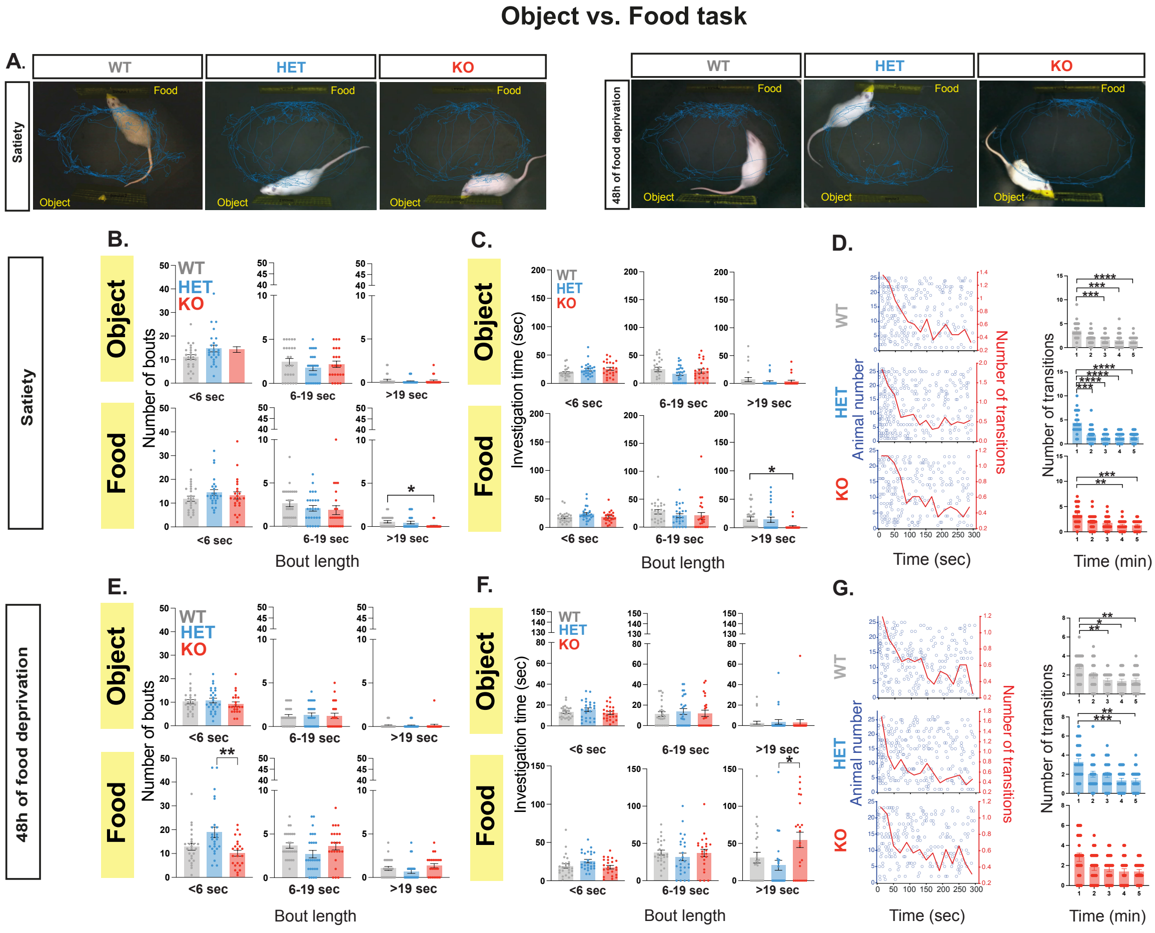

Extended Data Figure 8. A. A representative trace (from one rat per genotype) during the Object vs. Food task, at satiety (left) and after 48 hours of food deprivation (right). **B.** Mean combined number of investigation bouts (<6 sec), medium length bouts (6-19 sec), and long investigation bouts (>19 sec) during the Object vs. Food task during investigation of the moving object-containing (top) or food compartment (bottom) at satiety. **C.** Mean combined duration of short investigation bouts (<6 sec), medium length bouts (6-19 sec), and long investigation bouts (>19 sec) during the Object vs. Food task during investigation of the object-containing (top) or food compartment (bottom). **D**. Left, Transitions between the two compartments across time during the Object vs. Food task. Each punctum denotes the beginning of investigation of a new stimulus, and each row represents a single subject. The mean rate (using 20-s bins) is denoted by the red line (right red y-axis). Right, Mean pooled number of transitions between the two compartments

(object or food) over the 5-minute test period. **E-G**. Similar to B-D, but shows behavior during the Object vs Food task after 48 hours of food deprivation. WT, n=25; HET, n=26; KO, n=23. *$p<.05$, post hoc tests following the main effect. All error bars represent SEM. Detailed statistical data are provided as a Data file in Extended Data, Table 1.

Extended Data Figure 9.

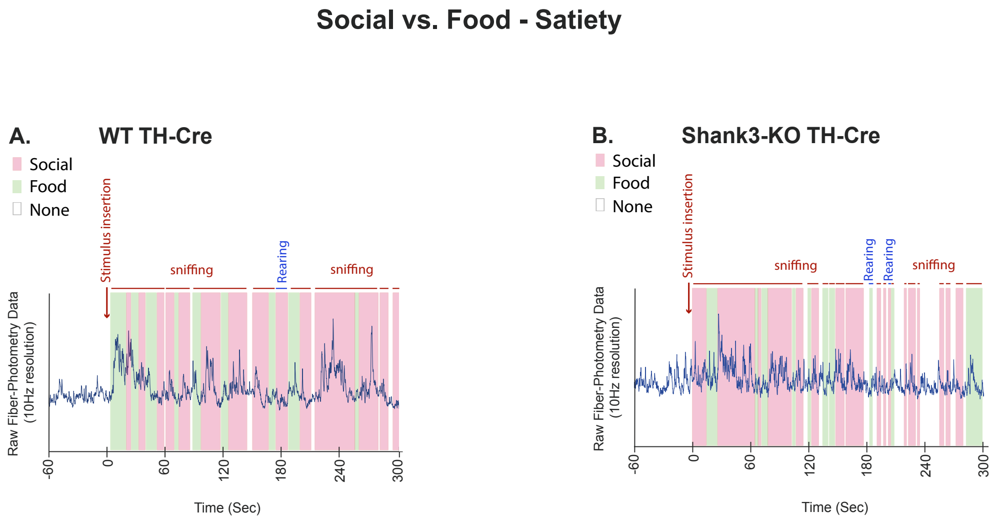

Extended Data Figure 9. A. Overlay of behavioral motifs and raw calcium events from a WT rat on the fiber photometry trace in VTA-DA neurons during the Social vs Food test at Satiety. **B.** Similar to A, but displaying the signal for a KO rat.

Extended Data Figure 10.

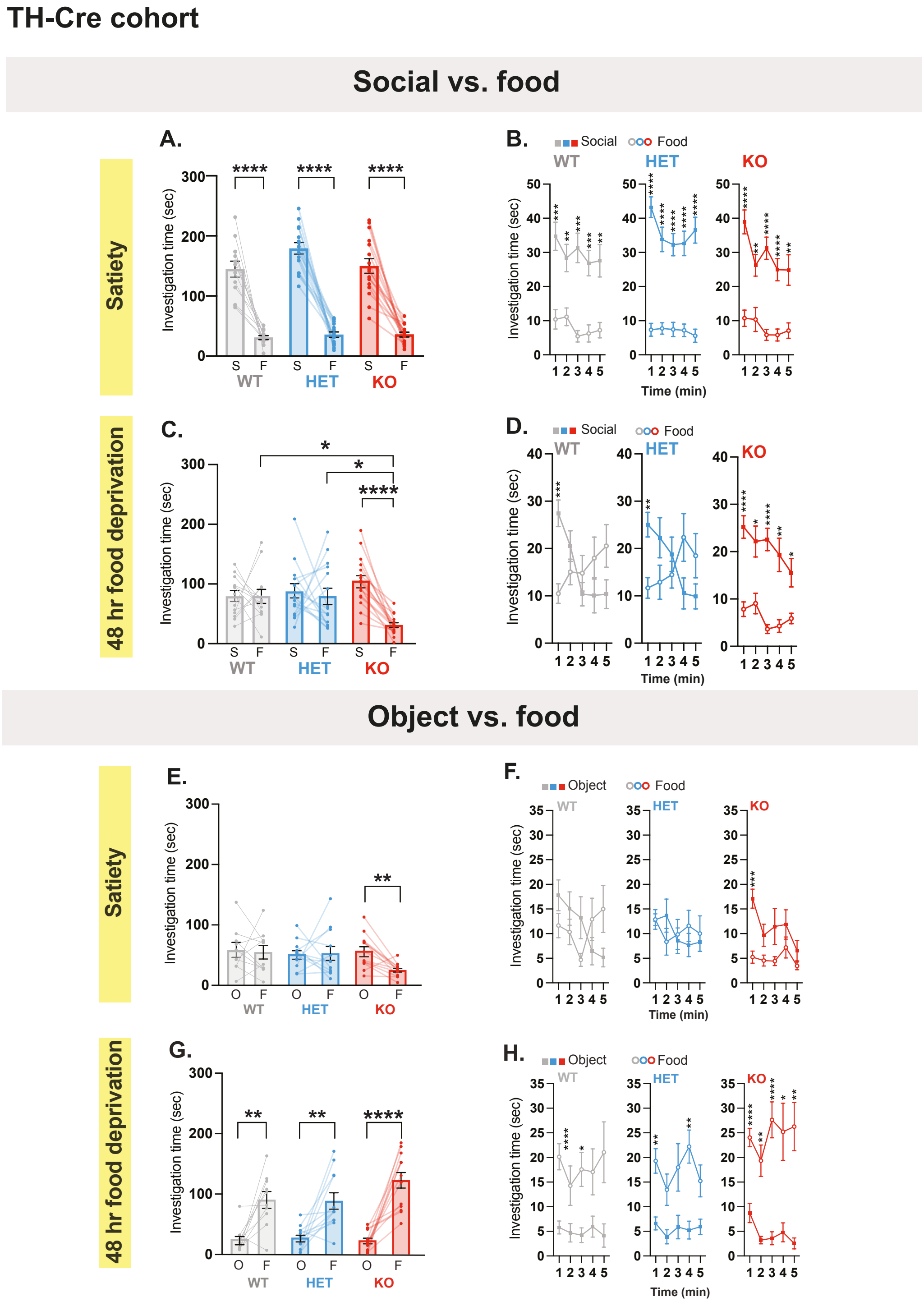

Extended Data Figure 10. A. Mean total investigation time of the social stimulus or the food compartment at satiety for the cohort of rats injected with TH-Cre and DIO-GCamp6 viruses, during testing on the Social vs. Food task. **B.** Mean total investigation time for the social stimulus

and the food compartment at satiety, averaged in 1-minute intervals over the 5-minute test period. **C and D.** Similar to A and B, but shows behavior after 48 hours of food deprivation. WT, n=13; HET, n=15; KO, n=16. **E-H.** Similar to A-D, but shows behavior during the Object vs Food task. WT, n=10; HET, n=12; KO, n=13. ****p<.0001, ***p<.001, **p<.01, *p<.05, post hoc tests following the main effect. All error bars represent SEM. Detailed statistical data are provided as a Data file.

Extended Data Figure 11.

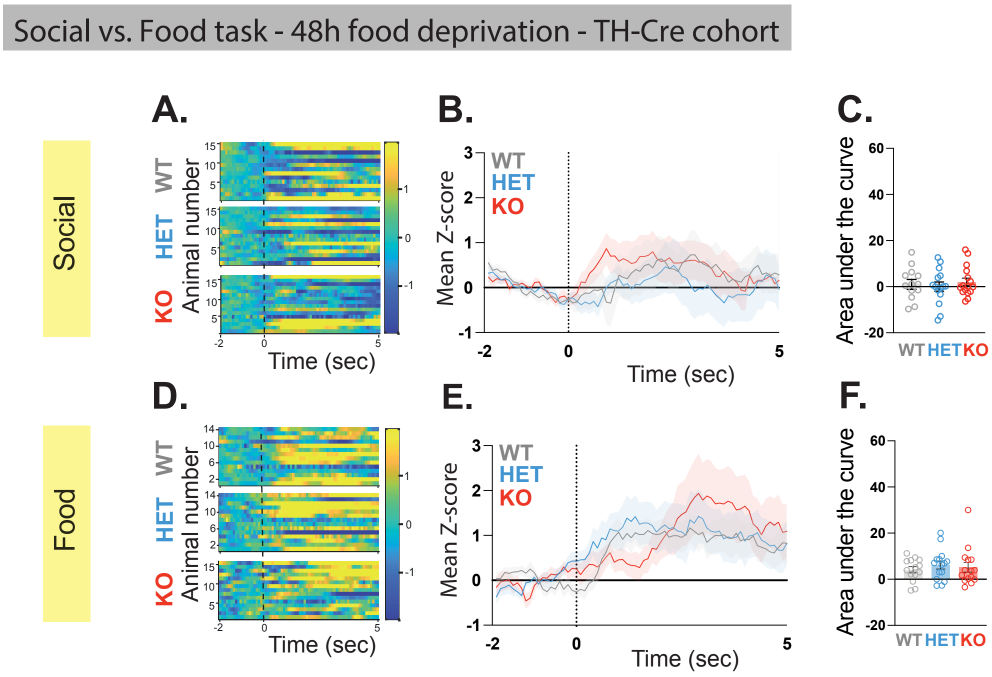

Extended Data Figure 11. A. Heat maps illustrate the change in fluorescence signals (dF/F) of GCaMP6 fiber photometry recordings of VTA-DA neurons, during the Social vs. Food task after 48 hours of food deprivation, from 2 seconds before to 5 seconds after each interaction bout for the social stimulus. Each row corresponds to one animal and comprises an average of signals from all interaction bouts during the 5-minute testing period. **B.** Average standardized VTA-DA photometry responses aligned to investigation onset of the social stimulus. **C.** The area under the curve, calculated from average standardized traces in (B). **D-F.** Similar to A-C, but show fiber photometry recording when the rats explore the food**.** WT, n=14; HET, n=14; KO, n=16.

Extended Data Figure 12.

GRAB-DA cohort

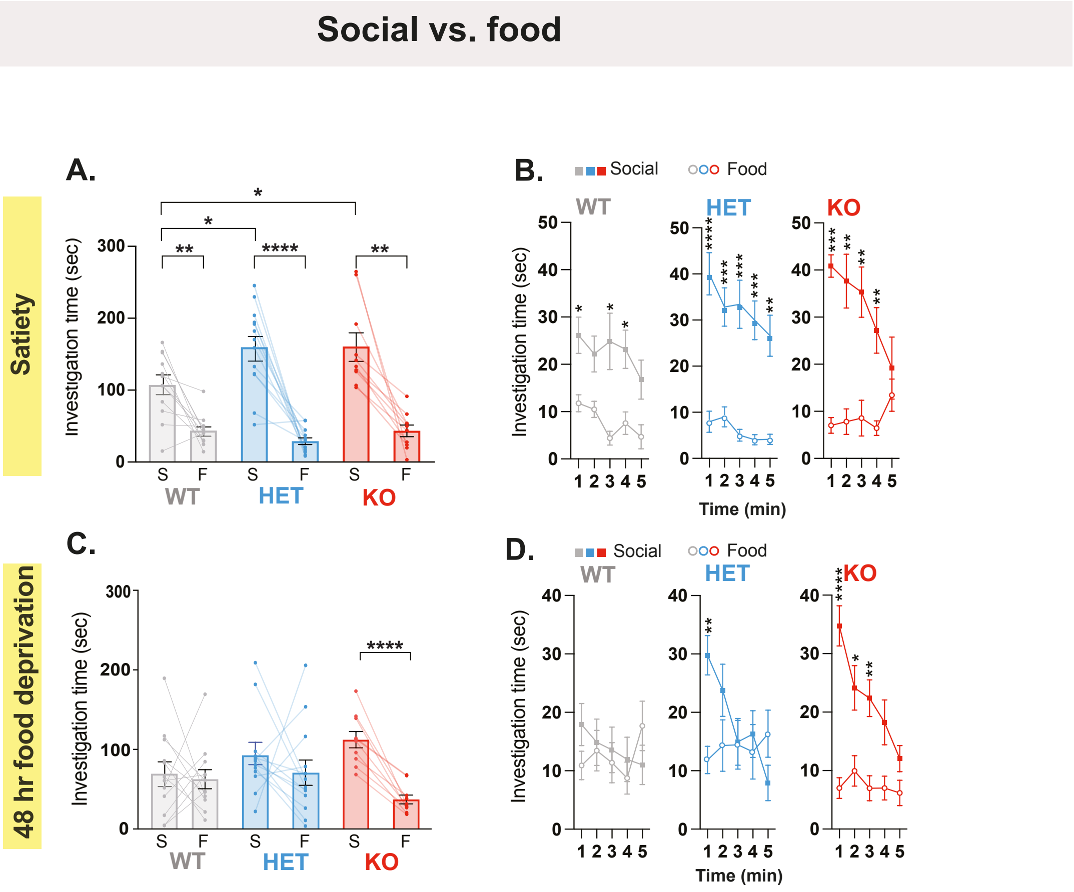

Extended Data Figure 12. A. Mean total investigation time of the social stimulus or the food compartment at satiety for the cohort of rats injected with $GRAB_{DA}$, during testing on the Social vs. Food task. **B.** Mean total investigation time for the social stimulus and the food compartment averaged in 1-minute intervals over the 5-minute test period. **C** and **D.** Similar to A and B respectively, but show behavior after 48 hours of food deprivation. WT, n=12; HET, n=13; KO, n=10. *****p*<.0001, ****p*<.001, ***p*<.01, **p*<.05, post hoc tests following the main effect. All error bars represent SEM. Detailed statistical data are provided as a Data file in Extended Data, Table 1.

Extended Data Figure 13.

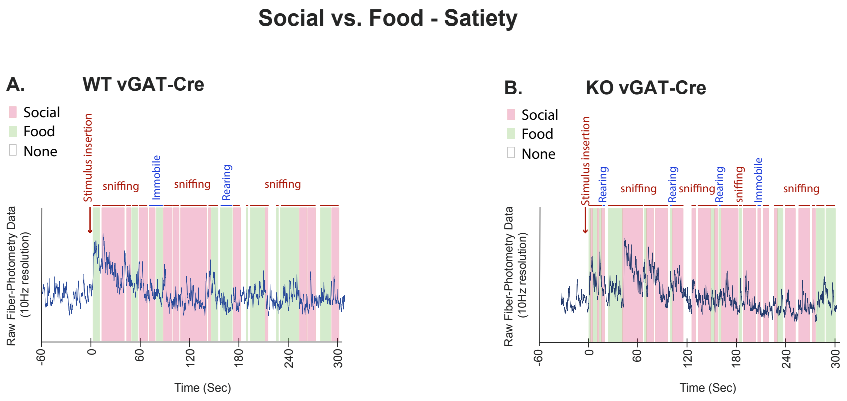

Extended Data Figure 13. A. Overlay of behavioral motifs and raw calcium events from a WT rat on the fiber photometry trace in VTA-vGAT neurons during the Social vs Food test at Satiety. **B.** Similar to A, but displaying the signal for a KO rat.

Extended Data Figure 14.

vGat cohort

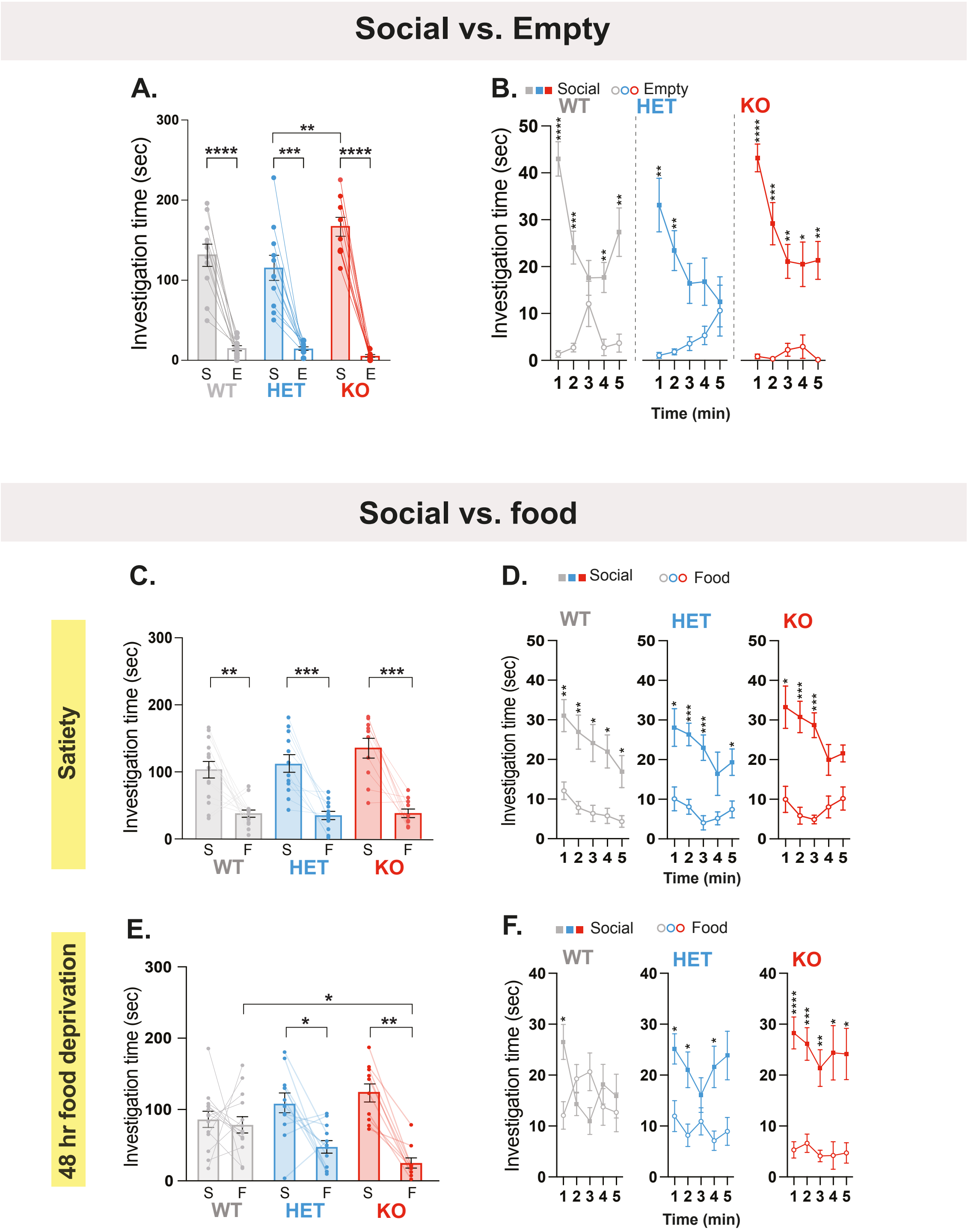

Extended Data Figure 14. A. Mean total investigation time of the social stimulus or the empty compartment for the cohort of rats injected with vGAT-Cre and DIO-GCamp6 viruses, during testing on the Social vs. Empty task at Satiety. **B.** Mean total investigation time for the social

stimulus and the empty compartment averaged in 1-minute intervals over the 5-minute test period. WT, n=11; HET, n=11; KO, n=10. **C** and **D.** Similar to A and B, respectively, but show behavior during the Social vs Food task at satiety (C) and after 48 hours of food deprivation (D). WT, n=14; HET, n=12; KO, n=10. ****p<.0001, ***p<.001, **p<.01, *p<.05, post hoc tests following the main effect. All error bars represent SEM. Detailed statistical data are provided as a Data file in Extended Data, Table 1.

Extended Data Figure 15.

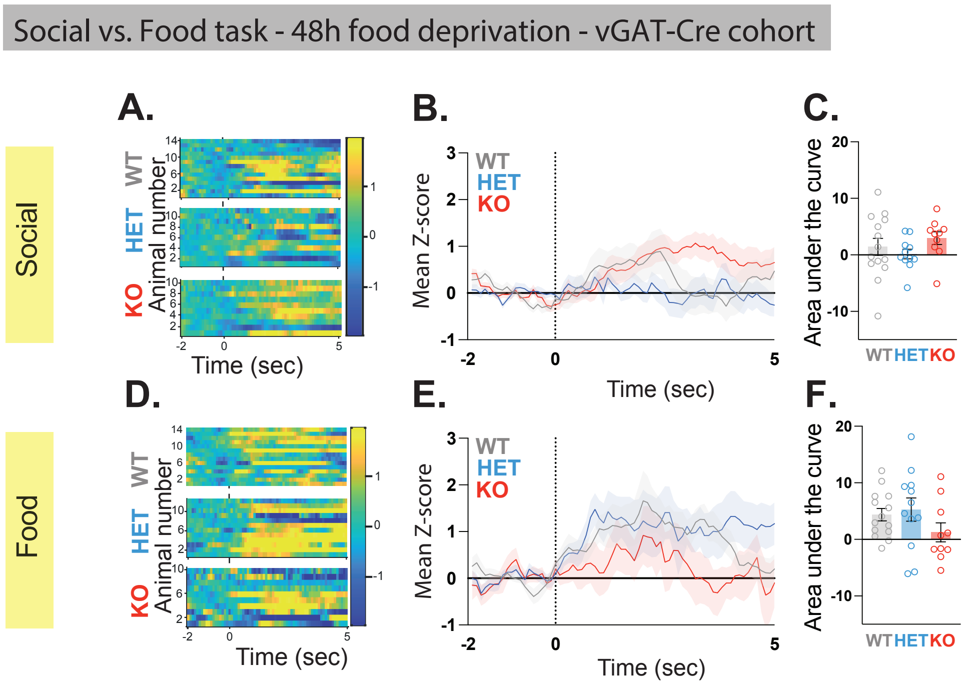

Extended Data Figure 15. A. Heat maps illustrate the change in fluorescence signals (dF/F) of GCaMP6 fiber photometry recordings of vGAT-DA neurons, during the Social vs. Food task after 48 hours of food deprivation, from 2 seconds before to 5 seconds after each interaction bout for the social stimulus. Each row corresponds to one animal and comprises an average of signals from all interaction bouts during the 5-minute testing period. **B.** Average standardized VTA-vGAT photometry responses aligned to investigation onset of the social stimulus. **C.** The area under the curve, calculated from average standardized traces in (B). **D-F.** Similar to A-C, but show fiber photometry recording when the rats explore the food**.** WT, n=14; HET, n=12; KO, n=10.

Extended Data Figure 16.

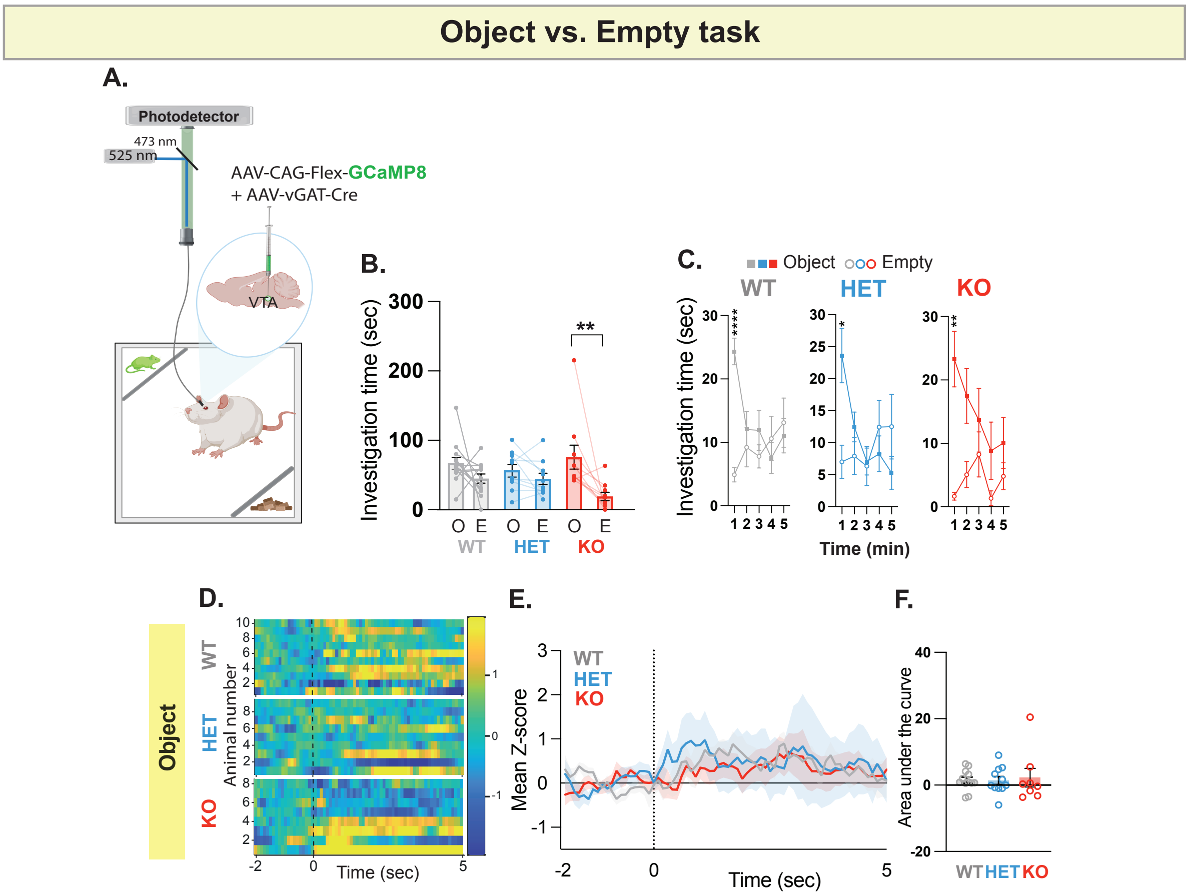

Extended Data Figure 16. A. A schematic of viral injection and fiber photometry setup in the VTA to record neural activity of VTA-GABAergic neurons. **B.** Mean total investigation time of the object stimulus or the empty compartment for the cohort of rats injected with vGAT-Cre and DIO-GCamp6 viruses, during testing on the Object vs. Empty task at Satiety. **C.** Mean total investigation time for the object stimulus and the empty compartment averaged in 1-minute intervals over the 5-minute test period. **D.** Heat maps illustrate the change in fluorescence signals (dF/F) from 2 seconds before to 5 seconds after each interaction bout with the object. Each row corresponds to one animal and comprises an average of signals from all interaction bouts during the 5-minute testing period. **E.** Average standardized VTA-vGAT photometry responses aligned to investigation onset of the object stimulus. **F.** The area under the curve, calculated from average standardized traces in (E). WT, n=13; HET, n=10; KO, n=10. ****p<.0001,

**$p<.01$, *$p<.05$, post hoc tests following the main effect. All error bars represent SEM. Detailed statistical data are provided as a Data file in Extended Data, Table 1.

Extended Data Figure 17.

Object vs. Food task

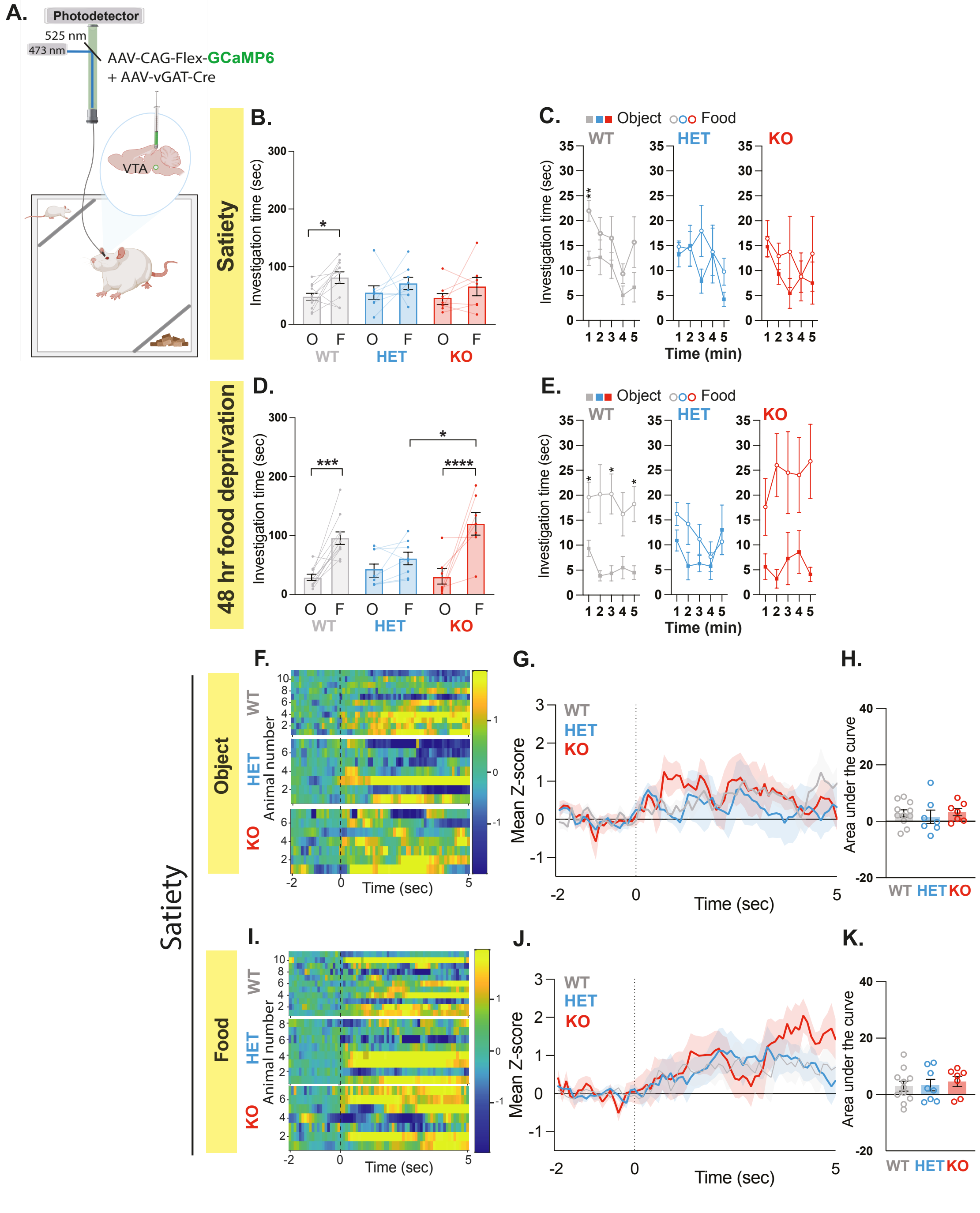

Extended Data Figure 17. A. A schematic of viral injection and fiber photometry setup in the VTA to record neural activity of VTA-GABAergic neurons. **B.** Mean total investigation time of the object stimulus or the food compartment at satiety for the cohort of rats injected with vGAT-Cre and

DIO-GCamp6 viruses, during testing on the Object vs. Food task. **C.** Mean total investigation time for the object stimulus and the food compartment averaged in 1-minute intervals over the 5-minute test period. **D** and **E.** Similar to B and C, respectively, but show behavior after 48 hours of food deprivation. **F.** Heat maps illustrate the change in fluorescence signals (dF/F) from 2 seconds before to 5 seconds after each interaction bout with the object. Each row corresponds to one animal and comprises an average of signals from all interaction bouts during the 5-minute testing period. **G.** Average standardized VTA-vGAT photometry responses aligned to investigation onset of the object stimulus. **H.** The area under the curve, calculated from average standardized traces in (G). **I-K.** Similar to F-H, but show fiber photometry signal for the food investigation. WT, n=11; HET, n=8; KO, n=7. $^{****}p<.0001$, $^{**}p<.01$, $^{*}p<.05$, post hoc tests following the main effect. All error bars represent SEM. Detailed statistical data are provided as a Data file in Extended Data, Table 1.
